# New Insights in Luminescence and Quenching Mechanisms of Ag_2_S Nanocrystals through Temperature-Dependent Spectroscopy

**DOI:** 10.1021/acs.jpclett.4c01439

**Published:** 2024-08-08

**Authors:** Jur W. de Wit, Irene Zabala-Gutierrez, Riccardo Marin, Adilet Zhakeyev, Sonia Melle, Oscar G. Calderon, Jose Marques-Hueso, Daniel Jaque, Jorge Rubio-Retama, Andries Meijerink

**Affiliations:** † Debye Institute for Nanomaterials Science, Utrecht University, 3584 CC Utrecht, The Netherlands; ‡ Departamento de Química en Ciencias Farmacéuticas, Universidad Complutense de Madrid, 28040 Madrid, Spain; § Nanomaterials for bioimaging group (nanoBIG), Facultad de Ciencias, Universidad Autónoma de Madrid, C/Francisco Tomás y Valiente 7, 28049 Madrid, Spain; ∥ Institute for Advanced Research in Chemical Sciences (IAdChem), Universidad Autónoma de Madrid, 28049 Madrid, Spain; ⊥ Institute for Sensors, Signals and Systems, Heriot-Watt University, EH14 4AS Edinburgh, U.K.; # Department of Optics, Complutense University of Madrid, E-28037 Madrid, Spain; ∇ Institute for Materials Science (ICMUV), University of Valencia, 46980 Valencia, Spain

## Abstract

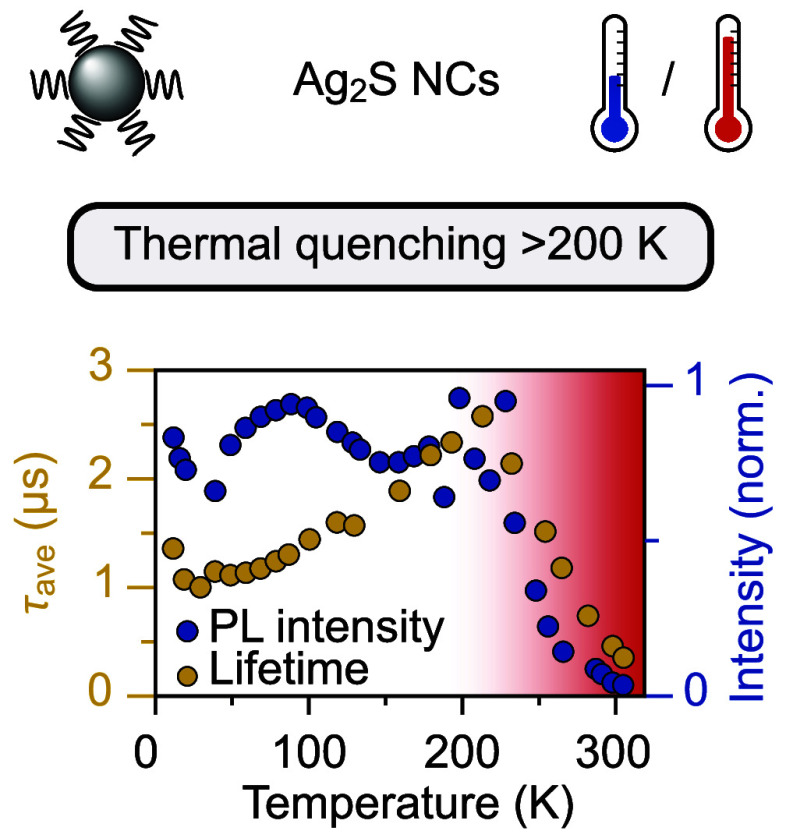

Bright near-infrared-emitting Ag_2_S nanocrystals (NCs) are used for in vivo temperature sensing relying on a reversible variation in intensity and photoluminescence lifetime within the physiological temperature range. Here, to gain insights into the luminescence and quenching mechanisms, we investigated the temperature-dependent luminescence of Ag_2_S NCs from 300 to 10 K. Interestingly, both emission and lifetime measurements reveal similar and strong thermal quenching from 200 to 300 K, indicating an intrinsic quenching process that limits the photoluminescence quantum yield at room temperature, even for perfectly passivated NCs. The low thermal quenching temperature, broadband emission, and multiexponential microsecond decay behavior suggest the optical transition involves strong lattice relaxation, which is consistent with the recombination of a Ag^+^-trapped hole with a delocalized conduction band electron. Our findings offer valuable insights for understanding the optical properties of Ag_2_S NCs and the thermal quenching mechanism underlying their temperature-sensing capabilities.

Semiconductor nanocrystals (NCs) are attractive for in vivo bioimaging and optical biosensing due to their small size and tunable optical properties.^1,2^ Particularly near-infrared (NIR) emitting NCs are interesting candidates because of the deep penetration depth achieved for this wavelength region, minuscule scattering, and the reduction of background autofluorescence produced by NIR excitation. Ag_2_S NCs have emerged as a prime choice in this context, since they feature low (cyto)toxicity^3,4^ and temperature-dependent optical properties ranging from room temperature (RT) to ∼60 °C which enable accurate luminescence thermometry.^5−7^ For sizes larger than 4.5 nm Ag_2_S NCs strongly absorb in the first biological window (750–900 nm),^8^ while their emission band falls within the second biological window (1000–1700 nm).^9^ However, the low photoluminescence quantum yields (QYs) reported for Ag_2_S NCs (typically below 1%) limit their performance.

Since the first reports on fluorescent Ag_2_S NCs in 2010,^10^ efforts to enhance their QY have been focused on improving the quality of the Ag_2_S core material and implementing effective surface treatments.^11−13^ Generally, Ag_2_S NCs in organic media are synthesized by the thermal decomposition of Ag-diethyldithiocarbamate (Ag-DDTC) as a silver and sulfur precursor in oleylamine and dodecanethiol (DDT), acting as both solvent and capping ligand. The compositional tuning achieved by changing the solvent ratio improved the QY of Ag_2_S NCs to roughly 2%, yielding Ag_2_S NCs with a more stoichiometric matrix.^14^ Further improvements were realized postsynthetically by sonochemical reaction or by ultrafast laser irradiation treatments in chloroform. The sonication results in the formation of small amounts of HCl that cause surface etching and give rise to less surface quenching sites, whereas during the ultrafast laser treatment a protective AgCl shell is formed.^15,16^ Both procedures result in QYs reaching 10%; the term superdots was coined for these highly luminescent Ag_2_S NCs. Despite these extensive efforts and the impressive improvement in efficiency, the photoluminescence QY of Ag_2_S NCs still lags behind that of other NIR-emitting NCs such as PbSe (QY ∼ 40%).^17^

A more in-depth comprehension of the origin of the NIR emission and the quenching mechanism is essential to gain more insight into the limitations and potential of Ag_2_S NCs. For small (<4.5 nm) Ag_2_S NCs, a size dependence of the emission maximum was observed, based on which the exciton Bohr radius for Ag_2_S was estimated to be around 2 nm.^18,19^ For NC sizes larger than 4.5 nm, the emission maximum stabilizes at ∼1200 nm, which is at a slightly lower energy than the bulk Ag_2_S bandgap energy of about 1.1 eV.^10,20^ In ref [^18^], sub-μs photoluminescence lifetimes were reported for a series of NCs with sizes ranging between 2.2 and 7 nm. Even though the emission maximum did not redshift above a size of 4.5 nm, the average lifetime lengthened.^18^ The current understanding of the photoluminescence properties of Ag_2_S NCs is primarily based on optical measurements between 0 and 60 °C, where strong temperature quenching and a redshift of the emission maximum are typically observed.^5,16^

It is surprising that in analyses of the photoluminescence properties of Ag_2_S NCs to date, there have only been a few reports that have included temperature-dependent optical measurements below 0 °C. Pereplitsa et al. observed a 40-fold decrease in photoluminescence (PL) intensity when varying from 80 to 300 K in 2.2 nm silica-coated Ag_2_S NCs. However, the origin of this decrease was not explained.^21^ To the best of our knowledge, temperature-dependent and time-resolved spectroscopy down to 10 K has not yet been reported, even though this can provide valuable information about the emission and quenching mechanisms.

In this paper, we report on the photoluminescence properties of bright Ag_2_S NCs between 10 and 300 K. Both photoluminescence intensity and time-resolved spectroscopy reveal that strong and reversible quenching occurs between 200 and 300 K for both DDT- and poly(ethylene glycol) (PEG)-capped Ag_2_S NCs. This indicates an intrinsic quenching mechanism which limits the RT QY to about 15%. Additionally, it plays a pivotal role in generating consistently reproducible temperature-dependent behavior in both emission intensity and lifetime, which is exploited in the application of Ag_2_S NCs for temperature sensing. Upon further cooling below 200 K, a remarkable temperature dependence of the average emission lifetime is observed and attributed to the thermal population of multiple dark and bright exciton states existing in Ag_2_S. These results contribute to a better understanding of the optical properties of Ag_2_S NCs and can aid in further optimizing Ag_2_S NCs for bioimaging and sensing applications.

We synthesized Ag_2_S NCs using a thermal decomposition method and subsequently subjecting them to a sonication step to enhance their PLQY, following the procedure outlined by Zabala-Gutierrez et al.^15^ This procedure results in dodecanethiol-terminated NCs (Ag_2_S-DDT) or, after a ligand exchange procedure also outlined in ref [^15^], in similarly sized pegylated NCs (Ag_2_S-PEG). Experimental details can be found in Supporting Information Section S1 describing the ligand exchange procedure and showing FTIR spectra before and after ligand exchange (Figure S1) confirming successful exchange. Figures 1a,b shows transmission electron microscope (TEM) images of both types of Ag_2_S NCs, with a diameter of 9 ± 1 nm for the Ag_2_S-PEG NCs and 9 ± 2 nm for the Ag_2_S-DDT NCs. In order to make Ag_2_S NCs biocompatible, they are often capped with PEG. We investigate and compare the properties of both Ag_2_S-PEG and Ag_2_S-DDT NCs to be able to distinguish effects related to the surface ligands from intrinsic photoluminescence properties of the Ag_2_S cores. To avoid differences due to solvent effects, all measurements on the Ag_2_S NCs are performed in chloroform (CHCl_3_).

**Figure 1:**
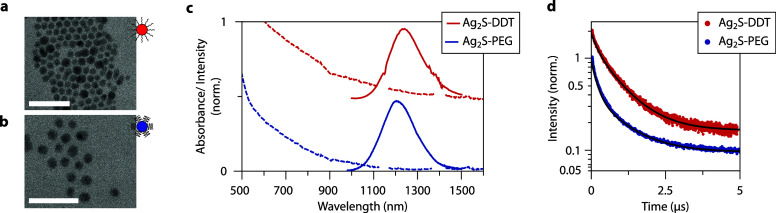
Transmission electron microscopy images and room temperature optical properties of Ag_2_S NCs capped with DDT and PEG. (a) TEM image of Ag_2_S-DDT NCs and (b) Ag_2_S-PEG NCs. Both scale bars are 50 nm. (c) Absorption (dashed) and emission spectrum (full, λ_exc_ = 520 nm) of Ag_2_S-DDT NCs and Ag_2_S-PEG NCs. (d) Luminescence decay curves of Ag_2_S-DDT NCs (λ_exc_ = 520 nm, λ_em_ = 1220 nm) and Ag_2_S-PEG NCs (λ_exc_ = 520 nm, λ_em_ = 1205 nm), recorded at 289 and 293 K, respectively. They are fitted with a double-exponential function represented by the black lines. The offset between the emission spectra (c) and decay curves (d) is added for clarity.

In Figure 1c, the RT absorption and emission spectra of Ag_2_S-DDT and Ag_2_S-PEG NCs are shown. The absorption spectrum consists of a featureless broad band with an onset at around 1200 nm. Chloroform C–H vibrational overtone absorptions around 1150 and 1400 nm are left out for clarity but are shown in the full spectra in Figure S2. No excitonic features are observed, which are typically present for semiconductor quantum dots in the quantum confinement regime. This observation is consistent with expectations, since ∼9 nm Ag_2_S NCs are not affected by quantum confinement effects, based on the exciton Bohr radius values for Ag_2_S (∼2 nm).^18,19^ Upon excitation at 520 nm, very similar emission spectra are observed with maxima around 1220 nm (for Ag_2_S-DDT NCs) and 1205 nm (for Ag_2_S-PEG NCs) and a full width at half-maximum (fwhm) of 140 meV (Ag_2_S-PEG NCs) and 150 meV (Ag_2_S-DDT NCs). The RT QY of these samples is 5.1 ± 0.7% for the Ag_2_S-DDT NCs and 1.6 ± 0.2% for the Ag_2_S-PEG NCs (details about the QY measurements can be found in Supporting Information Section S3). The lower QY for the Ag_2_S-PEG NCs has been ascribed to a poorer surface passivation or a higher concentration of surface quenching centers induced during functionalization with PEG, resulting in increased quenching caused by surface-related traps.^14^

To gain further insight into the RT optical properties, we recorded luminescence decay curves for both types of Ag_2_S NCs (Figure 1d). Both exhibit nonexponential decays, indicating the presence of multiple de-excitation pathways. Decay curves were fitted with a two-exponential function which results in an average lifetime
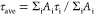
1where *A*_i_ is the intensity at time 0 and τ_i_ is the decay time of component i. The values for τ_ave_ at RT are 0.63 μs for the Ag_2_S-DDT NCs and 0.46 μs for the Ag_2_S-PEG NCs. A two-exponential function gives a good description of the experimentally observed decay behavior (Figure S4) and can be used to quantify changes in decay behavior. However, it is important to realize that decay rates vary for different Ag_2_S NCs as a result of many different de-excitation rates originating from variations in surface trap concentrations. The fit to a biexponential function should not be understood as there being only two different decay rates for different Ag_2_S NCs. The two lifetimes provide a good description of the multiexponential decay kinetics of the ensemble of Ag_2_S NCs. The observation of a nonexponential decay is in line with previous reports, where the origin of nonexponential decay has been attributed to the quenching of the emission by surface traps.^15,16^ It is interesting to observe that even for the highly efficient Ag_2_S NCs the decay is nonexponential. The decay times show a clear correlation with the QY. While for the highly efficient NCs an ∼2 μs average lifetime was observed at RT, the decay curves for less efficient Ag_2_S NCs is much shorter, in the ∼100 ns time range.^16^ The lifetimes measured here for the Ag_2_S NCs are shorter than 2 μs, in agreement with a QY lower than that for the best Ag_2_S NCs (up to 10% QY). The shorter average lifetime of the Ag_2_S-PEG compared to Ag_2_S-DDT NCs agrees with their lower QY value. The faster decay for the less efficient (lower QY) Ag_2_S NCs can be qualitatively explained by additional nonradiative (surface) quenching pathways. However, there is no linear dependence between the QY and average lifetime. This reflects that the luminescence decay curves do not capture all nonradiative processes. For instance, very fast direct quenching can diminish the QY, yet this effect may be too fast to capture and can be obscured in decay curves when measured with an insufficient time resolution.

Previously reported temperature-dependent behavior of Ag_2_S photoluminescence above RT shows marked and reversible quenching resulting in a drop in both luminescence intensity and lifetime.^5^ To better understand the quenching, we investigated the temperature-dependent emission spectra of Ag_2_S NCs within the temperature range from 10 to 310 K. As the temperature decreases, the emission peak position of Ag_2_S-PEG NCs undergoes a blueshift from 1205 nm at RT to 1080 nm at 16 K, corresponding to an emission peak shift of 120 meV for the Ag_2_S-PEG NCs (Figure 2a, Figure S5b). A similar peak shift of 125 meV is observed for the Ag_2_S-DDT NCs (Figure 2b, Figure S6b). This behavior can be described by the Varshni expression for the relationship between the band gap energy and temperature in semiconductors. The peak shift is larger than the shift observed in, e.g., CdSe NCs (70 meV) and CuInS_2_ NCs (50 meV).^22,23^ In addition to the blueshift, the emission band also narrows with decreasing temperature, showing a full width at half-maximum (fwhm) reduction to about 100 meV for both the Ag_2_S-PEG and Ag_2_S-DDT NCs (Figure S5b, Figure S6b). The fwhm at 10 K is considerably larger than the bandwidths observed for II–VI or IV–VI QDs. For example, in the case of CdSe QDs, the fwhm is reduced to ∼40–80 meV. This reduction depends on the polydispersity of particle size as a consequence of the well-known variation in emission wavelength with size due to the effects of quantum confinement. For single CdSe quantum dots, extremely narrow emission lines (<1 meV) are observed at 10 K, which is expected for the recombination of a delocalized conduction band (CB) electron and a delocalized valence band (VB) hole. Because of the delocalized nature of both charge carriers, the lattice relaxation involved in the optical transition is small, resulting in narrow emission lines (weak electron–phonon coupling). For 10 nm Ag_2_S NCs there is no inhomogeneous broadening, and narrow band emission is expected at the band gap energy for CB electron with VB hole recombination. The observed large fwhm for the Ag_2_S emission at 10 K suggests a much larger electron–phonon coupling. Analysis of the temperature-dependent broadening of the emission band and the peak shift confirms the strong electron–phonon coupling for emission transition (Supporting Information, section S6). The broad emission band can be explained by a luminescence mechanism, where fast hole trapping occurs by Ag^+^. This results in the formation of Ag^2+^, with a formal 2+ charge of Ag but in fact involving a charge redistribution, including the S^2–^ ligands and a shortening of the Ag–S distance. Hole trapping is followed by radiative recombination with a CB electron, resulting in emission that involves strong local lattice relaxation.^24^ A similar mechanism has been reported for broad band emission for various Ag- and Cu-doped and Cu-based semiconductor nanocrystals.^25^

**Figure 2:**
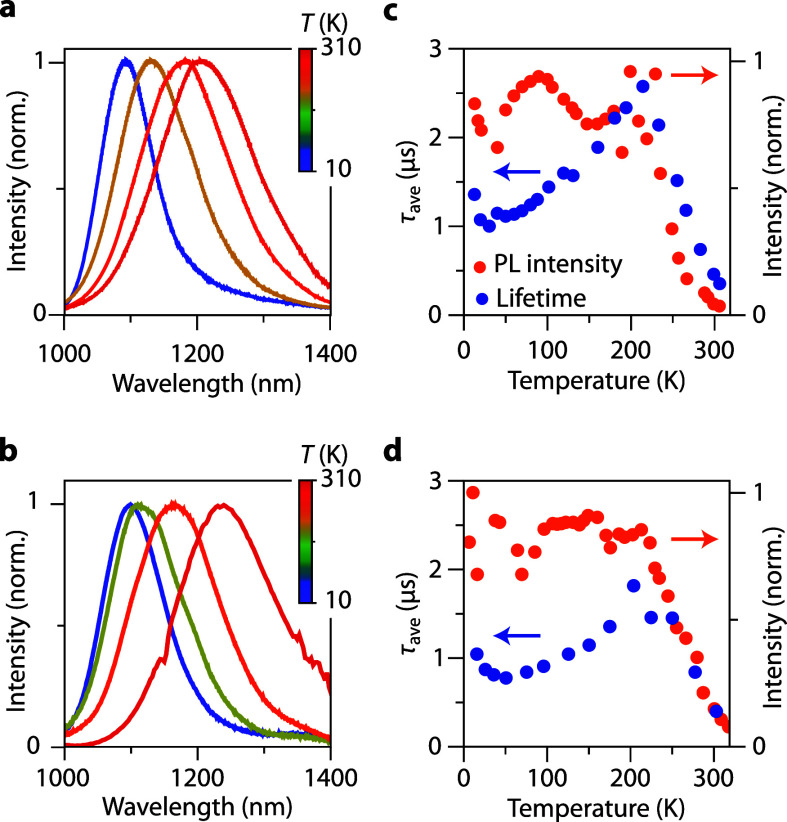
Temperature-dependent emission spectroscopy on Ag_2_S NCs. (a) Emission spectra of Ag_2_S-PEG NCs recorded at (blue to red) 13, 128, 220, and 289 K. (b) Emission spectra of Ag_2_S-DDT NCs recorded at (blue to red) 16, 120, 175, and 293 K. (c) Integrated photoluminescence intensity plotted as a function of temperature (orange) together with the average lifetime as a function of temperature (blue) for Ag_2_S-PEG NCs. (d) Same as in panel (c) but for Ag_2_S-DDT NCs. All spectra and decay curves were recorded with λ_exc_ = 520 nm; the emission maximum wavelength was used to record decay curves.

The emission spectra in Figure 2a,b are plotted after normalization to observe the peak shift and changes in the emission bandwidth more clearly. To determine the temperature dependence of the emission intensity, the integrated emission spectra were calculated and are plotted in Figure 2c,d for the DDT and PEG-capped Ag_2_S NCs as a function of temperature. Both NCs show a sharp increase of intensity upon cooling from 300 to 200 K. The PEG-capped NCs exhibit a 15-fold increase, whereas the DDT-capped NCs show a 9-fold increase. Below 200 K the intensity remains constant for both samples. Because the measurements are performed on Ag_2_S NCs dispersed in chloroform, we have to correct for the intensity shift caused by the solidification of chloroform around 210 K. Details about this correction, including the uncorrected intensity values as a function of temperature, can be found in the Supporting Information Section S5. The fluctuations in intensity are relatively large between 10 and 200 K. We do not attribute these variations in emitted intensity to thermal quenching but to artifacts related to several factors, such as changes in sample alignment, condensation on the cryostat windows, excitation source intensity variations, change on collection efficiency due to the phase transition in the solvent, or temperature-dependent absorption strength.^26^

All of these factors make it challenging to study thermal quenching by recording the emission intensity as a function of temperature. A complementary and insightful approach for investigating thermal quenching involves measuring the emission lifetime as a function of the temperature. For a constant radiative decay rate, quenching through thermally activated nonradiative decay will result in a shortening of the emission lifetime along with a drop in emission intensity. Note that variations in intensity due to artifacts discussed above do not affect the temperature-dependent luminescence lifetime as the decay kinetics are an inherent property of luminescent species and the dynamics are unaffected by external factors that cause changes in integrated emission intensity. Therefore, we measure the luminescence lifetimes as a function of temperature for the Ag_2_S-PEG and Ag_2_S-DDT NCs between 10 and 310 K. Nonexponential decay curves were recorded at all temperatures. In Figure 3a selection of decay curves recorded at different temperatures at the maximum of the emission band is shown. The decay curves were fitted with biexponential functions to determine the average decay time τ_ave_ using eq 1. Upon cooling from RT to 200 K the average lifetime increases sharply and closely follows the increase in emission intensity for both samples (Figure 2c,d). For the Ag_2_S-PEG NCs τ_ave_ increases from 0.46 μs at 300 K to 2.6 μs at 215 K, while for Ag_2_S-DDT NCs τ_ave_ increases from 0.63 to 1.87 μs at 203 K. This corresponds to a 5.6-fold increase and a 3-fold increase in the average lifetime within this temperature range. The steep and consistent drop in lifetime and emission intensity between 200 and 300 K for both Ag_2_S-DDT and Ag_2_S-PEG NCs indicates that the temperature dependence of emission lifetime and intensity that is used for luminescence thermometry is based on an intrinsic luminescence quenching process that starts at 200 K and continues above RT.^6,7,16^ The large ∼10-fold decrease in intensity and ∼5-fold decrease in decay time upon heating from 200 to 300 K shows that the emission is already strongly quenched at 300 K. The strong intrinsic quenching at RT shows that the highest QY that can be obtained for Ag_2_S NCs is limited, consistent with the observation that, in spite of considerable effort, the QY at RT for the best Ag_2_S NCs never exceeds 10%. The observation of quenching at relatively low temperatures (between 200 and 300 K) can be explained by an optical transition with strong electron–phonon coupling, consistent with the mechanism for the emission proposed above, viz., recombination of a trapped hole with a delocalized CB electron. In case of strong phonon coupling, quenching by thermally activated crossover to the ground state occurs at relatively low temperatures, especially for long wavelength emission.^27^ Previously, also for other semiconductor NCs showing broadband self-trapped exciton emission, luminescence quenching below RT has been reported and been used for temperature sensing.^28,29^ Interestingly, the present results show that Ag_2_S NCs are also promising for temperature sensing below 300 K.

**Figure 3:**
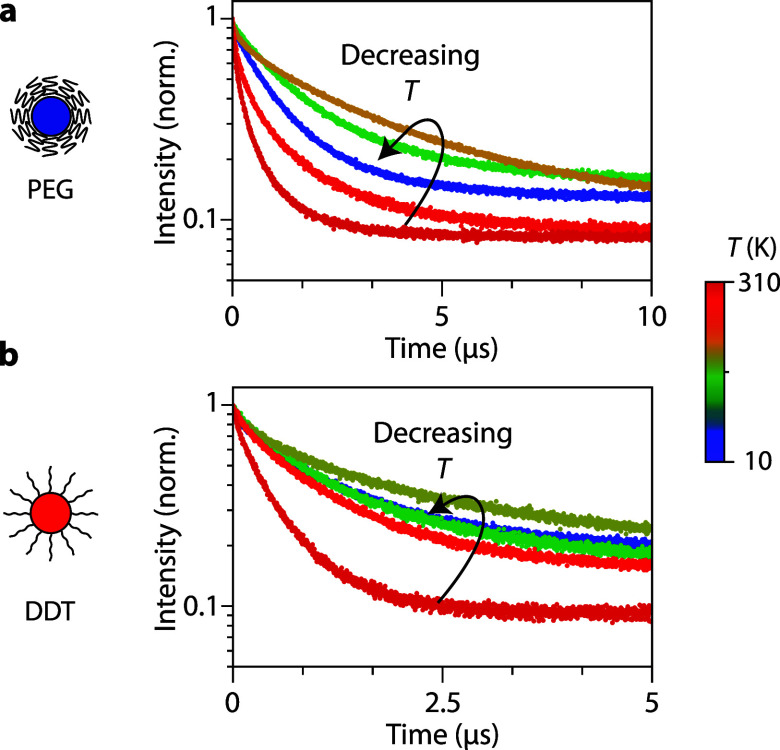
Temperature-dependent luminescence decay curves of Ag_2_S NCs. (a) Luminescence decay curves of Ag_2_S-PEG NCs at (blue to red) 20, 131, 195, 284, and 307 K. (b) Decay curves for Ag_2_S-DDT NCs recorded at 15, 131, 195, 285, and 308 K. All decay curves were recorded with excitation at 520 nm and emission at the maximum of the emission band.

To further corroborate the mechanism for NIR emission from Ag_2_S NCs, we examined the shape of the luminescence decay curves. A representative selection in Figure 3a,b depicts decay curves down to 10 K. More decay curves with overlaid fits can be found in Figure S7. Similar to the decay curves measured at RT and the ones reported in the literature, all the decay curves remain nonexponential. This observation suggests that the presence of multiple decay rates is characteristic of emission from Ag_2_S NCs. The nonexponential decay primarily arises from variations in nonradiative decay rates due to differences in (surface) defects quenching the emission for different Ag_2_S NCs. However, even for the most efficient Ag_2_S NCs still nonexponential decay is observed. Similar temperature-invariant nonexponential decay is observed for CuInS_2_ NCs and Ag-doped semiconductors like CdSe.^22,30^ In the case of Ag_2_S, depending on the exact location for trapping of the hole by Ag^+^, the overlap with the delocalized electron wave function will vary. Faster recombination is expected for a hole trapped in the center of the NC and slower recombination for trapping at the edge, where the electron wave function has a lower amplitude, resulting in a lower recombination probability. This is an additional explanation for the nonexponential behavior that is consistent with a luminescence mechanism of recombination of a trapped hole and a CB electron.

Surprisingly, below 200 K the average lifetime for both Ag_2_S-PEG and Ag_2_S-DDT NCs shortens upon further cooling to ∼50 K, where the average lifetime of Ag_2_S-PEG NCs drops to 1 μs and for Ag_2_S-DDT NCs drops to 0.8 μs. While the average lifetime decreases, the emission intensity remains constant. This suggests that, in contrast to the thermal quenching above 200 K, there is no change in the efficiency of the nonradiative process; instead, there is a change in radiative decay rates. Further reducing the temperature from 50 to ∼10 K reveals another increase in the average lifetime to 1.4 and 1.1 μs for Ag_2_S-PEG NCs and Ag_2_S-DDT NCs, respectively. It is noteworthy that despite differences in the observed average lifetime between Ag_2_S-PEG and Ag_2_S-DDT NCs, the thermal quenching (>200 K), lifetime shortening (50–200 K), and lifetime lengthening (<50 K) ranges are consistent for the two types of Ag_2_S NCs.

The unusual shortening in emission lifetime upon cooling below 200 K and the increase in lifetime below 50 K can be explained by the presence of thermally coupled excited states, from which radiative decay is possible. These states are a lowest energy dark state, a higher energy bright state, and again a dark state at even higher energies. In Figure 4, we schematically illustrate the behavior of this thermally coupled energy-level system at various temperatures. In Figure S8, we divide the temperature-lifetime plot into temperature regions corresponding to the three situations depicted in Figure 4. The lengthening of the lifetime upon cooling from 50 to 0 K is commonly observed in (chalcogenide) semiconductor such as CdSe, PbSe, and CuInS_2_ NCs and is attributed to freezing in all population in a lowest-energy “dark” exciton state separated by Δ*E*_1_ from a higher-energy bright state.^31^ Decay from this dark state is characterized by a longer radiative lifetime due to its spin-forbidden nature. For CdSe and PbSe QDs the slow (μs) decay component is determined by the weighted average population of the dark and bright states and has a single-exponential character, and an increasing decay rate (shorter lifetime) is observed upon raising the temperature.^32,33^ The temperature dependence of the decay rates makes it possible to determine Δ*E*_1_ and the lifetime of the low-energy dark state. Unfortunately, the inherent nonexponential decay in Ag_2_S NCs makes it difficult to quantitatively analyze the decay curves to determine Δ*E*_1_. The increase in lifetime between 50 and 200 K is unusual, as luminescence lifetimes typically decrease with temperature due to thermally activated nonradiative decays. The significant lengthening of the emission lifetime can be explained by the presence of additional higher-energy dark states, which become increasingly populated at elevated temperatures. In the proposed energy-level structure, the higher-lying energy state would need to have a higher degeneracy than the bright state to explain the strong increase in the decay time. Additional experiments and excited-state DFT calculations could offer further insights into the electronic structure of excitons in Ag_2_S NCs, to confirm the current explanation for the unusual increase in lifetime observed between 50 and 200 K. It is noteworthy that a lengthening of decay time has also been observed for CdSe quantum dots just above RT and consistent with theoretical energy-level calculations showing a lowest-energy dark state and a higher-energy bright state followed by even higher-energy dark states, analogous to the energy-level scheme in Figure 4.^34−36^

**Figure 4:**
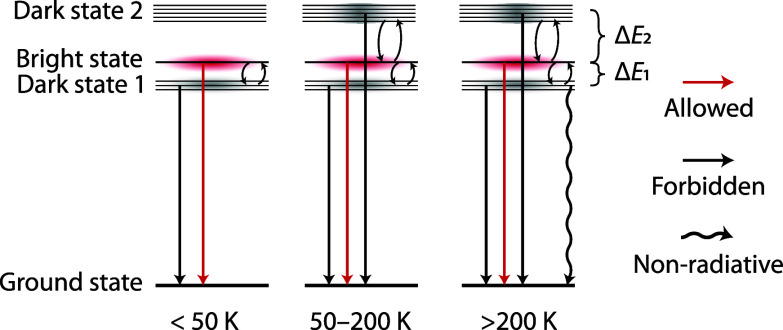
Proposed electronic structure of the emissive excited states in Ag_2_S NCs. At 0 K, only the lowest dark excited state is populated with slow decay because of the forbidden nature of the emission transition. At 0–50 K, the bright state is thermally populated as *k*_B_*T* > Δ*E*_1_ giving rise to faster decay from bright state. Upon further raising the temperature, the higher-energy dark state is populated and the forbidden nature of this transition causes lengthening of the emission lifetime (>50 K) until it rapidly drops because of thermal quenching (>200 K).

The luminescence and quenching mechanisms for Ag_2_S NCs with a diameter of ∼9 nm and capped with either DDT or PEG were investigated. At room temperature, a featureless absorption band starting in the NIR and a broad emission band around 1220 nm are observed in line with earlier reports on these bright NIR-emitting NCs. For the first time, the temperature-dependent photoluminescence properties of Ag_2_S NCs are reported in the temperature range from 10 to 310 K. Both temperature-dependent emission and time-resolved spectroscopy reveal strong and reversible thermal quenching between 200 and 300 K in both PEG- and DDT-capped Ag_2_S NCs. This intrinsic luminescence quenching is held responsible for the previously reported temperature-sensing capabilities for Ag_2_S NCs between RT and 60 °C based on a decrease in intensity and shortening of emission lifetime with temperature. It also limits the RT quantum yield of Ag_2_S NCs to ∼15%. Below 200 K the emission intensity remains constant, while the average lifetime initially shortens upon cooling to 50 K before lengthening again with further cooling below 50 K. To explain the remarkable variation in luminescence lifetime with temperature, we propose an energy-level scheme for Ag_2_S NCs with three thermally coupled excited states. Finally, based on the characteristic broad emission band, μs radiative lifetime, low thermal luminescence quenching temperature, Stokes-shifted emission and multiexponential decay, even at 10 K, we propose that the NIR emission originates from recombination of a delocalized conduction band electron with a hole localized on Ag^+^, similar to the recombination mechanism proposed for Ag-doped CdSe and CuInS_2_ NCs.

## Supporting Information Available

Experimental methods for synthesis and characterization, uncorrected absorption spectra, QY measurements, comparison of fitting procedures for time-resolved emission data, all temperature-dependent emission spectra with analysis, all time-resolved data with fitting fitting and comparison with three-level excited state model (PDF)
Transparent Peer Review report available (PDF)
