# Supplementary material for: New Insights in Luminescence and Quenching Mechanisms of Ag2S Nanocrystals through Temperature-Dependent Spectroscopy
Source: J Phys Chem Lett. 2024 Aug 8;15(33):8420–6. doi: 10.1021/acs.jpclett.4c01439 (PMC11345845; doi:10.1021/acs.jpclett.4c01439)
Supplement: Supplementary file 1 — jz4c01439_si_001.pdf [file jz4c01439_si_001.pdf]

Supporting Information for:

New Insights in Luminescence and Quenching Mechanisms of Ag_2S Nanocrystals Through Temperature-Dependent Spectroscopy

Jur W. de Wit[1], Irene Zabala-Gutierrez[2], Riccardo Marin[3,4,5], Adilet Zhakeyev[6], Sonia Melle[2], Oscar G. Calderon[2], Jose Marques-Hueso[7], Daniel Jaque[3,4,5], Jorge Rubio-Retama[5], Andries Meijerink[1]

1. Debye Institute for Nanomaterials Science, Utrecht University, 3584 CC Utrecht, The Netherlands
2. Departamento de Química en Ciencias Farmacéuticas, Universidad Complutense de Madrid, Madrid 28040, Spain
3. Nanomaterials for bioimaging group (nanoBIG), Facultad de Ciencias, Universidad Autónoma de Madrid, C/ Francisco Tomás y Valiente 7, Madrid 28049, Spain
4. Institute for Advanced Research in Chemical Sciences (IAdChem), Universidad Autónoma de Madrid, 28049 Madrid, Spain
5. Instituto Nicolás Cabrera, Universidad Autónoma de Madrid, 28049 Madrid, Spain
6. Institute for Sensors, Signals and Systems, Heriot-Watt University, EH14 4AS, Edinburgh, UK
7. Institute for Materials Science (ICMUV), University of Valencia, 46980, Valencia, Spain

Contents

Section S1. Experimental 2

Section S2. Absorption for Ag_2S-DDT and Ag_2S-PEG NCs 4

Section S3. Photoluminescence quantum yield measurements on Ag_2S NCs 5

Section S4. Time-resolved spectroscopy and fitting of the decaycurves 7

Section S5. Temperature-dependent emission spectra of Ag_2S NCs 8

Section S6. Temperature-dependent decay measurements 11

Section S1. Experimental

Chemicals

Silver nitrate (99.9%), sodium diethyldithiocarbamate (NaDDTC) (ACS reagent grade), oleylamine (70%) (OLAM), 1-DDT (≤98%), and toluene (99.5%) were purchased from Sigma-Aldrich. Heterofunctional methoxy PEG thiol (HS-PEG-OMe) with different molecular weights (MW = 750, 2000, 5000 $g·mol^{-1}$) were acquired from RAPP Polymere and used without further purification. Chloroform ($CHCl_3$, 99.6%) and ethanol absolute pure (99.8%) were purchased from PanReac AppliChem. Anhydrous tetrachloroethylene (TCE) (>99%) were obtained from Sigma Aldrich.

Synthesis of Ag_2S-DDT NCs

The Ag_2S NCs were synthesized based on a protocol described in ref [1]. In brief, AgDDTC was prepared by combining 200 ml of 0.125M $AgNO_3$ with 200 ml of 0.125 M NaDDTC. The AgDDTC precipitate was collected and vacuum dried at 60°C.

For a typical synthesis, 25 mg AgDDTC, 2.5 ml OLAM and 2.5ml DDT were combined in a 2-neck flask and degassed under vacuum and sonication. The flask was then filled with N_2 atmosphere and heated to 185°C using a preheated oil bath. After heating for 1 h at 185°C the flask was left to cool down, after which ethanol was added and the mixture was centrifuged at 10 000 *g* for 10 min to precipitate the Ag_2S NCs. This washing step was repeated twice before the NC were stored in 10 ml $CHCl_3$ with a final concentration of ~ 1mg/ml.

Sonication procedure

For the sonication step a Branson Sonifier 250 was used at 20W operating power with a duty cycle of 10%.[1] The horn was placed in a 10 ml vial containing 5 ml of a 0.3 mg ml^{-1} Ag_2S NC in $CHCl_3$ solution. The vial was placed in an ice bath to avoid heating up.

Ligand exchange to Ag_2S-PEG NCs

For the ligand exchange, 2 mg of PEG was added to 1 mg of sonicated Ag_2S NCs dispersed in 1 ml $CHCl_3$. The reaction was stirred vigorously for 30 min after which hexane was added to destabilize the dispersion. The precipitate was collected and redispersed in $CHCl_3$. The incorporation of the PEG-SH molecules on the surface of the nanoparticles is driven by the sonochemical treatment carried out prior to the incorporation of the PEG molecules. This process produces an etching reaction on the surface of the nanoparticles that reduces the number of DDT molecules anchored on the surface and renders NPs with pristine regions that can be functionalized with the PEG moieties. A complete description of the process can be found in Ref. [1]. In addition, to confirm the success of the PEG functionalization Figure S1 shows FTIR spectra of DDT and PEG functionalized nanoparticles. Here, we can observe that the incorporation of the PEG-SH modifies the FTIR spectrum of the nanoparticles, exhibiting bands that are specific of PEG molecules like the C-O stretching vibration at 1091 cm^{-1} and that does not appear in DDT functionalized nanoparticles. For the PEG-capped NCs we observe the CH_2 stretching vibrations at 2843 cm^{-1}. In addition, bands at 1478 and 1442 cm^{-1} can be assigned to the bending mode of the C-H group, while the bands that appeared at 1279 and 1229 cm^{-1} could be attributed to the C-H twisting vibrations. By contrast, the DDT functionalized nanoparticles exhibits a completely different FTIR spectrum that features sharp bands at 2922 and

2852 cm^{-1}, which can be assigned to the asymmetric methyl stretching and asymmetric/symmetric methylene stretching modes respectively. In summary, the FTIR spectra demonstrate clearly the incorporation of the PEG moieties and the success of the nanoparticle functionalization.

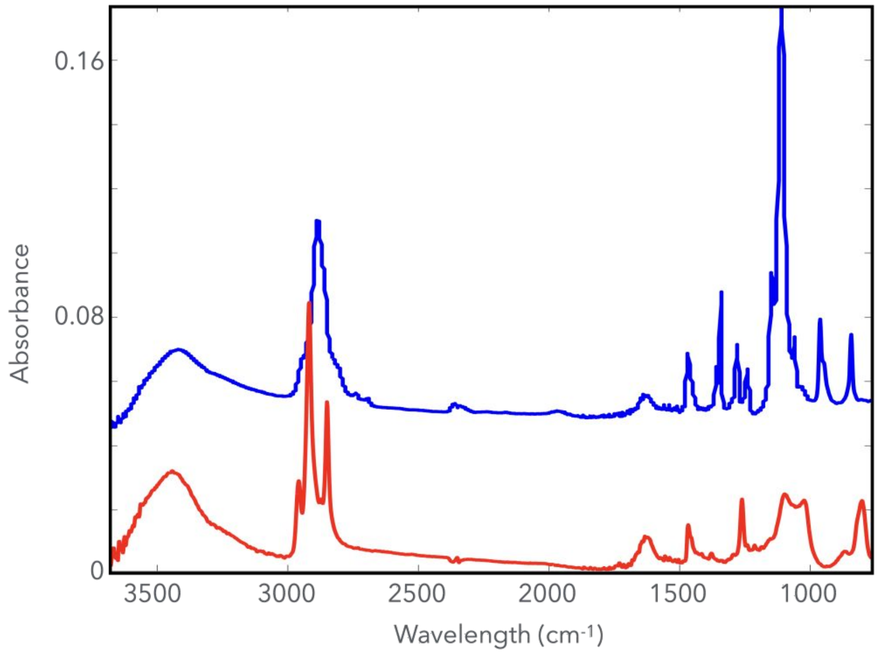

Figure S1 | FTIR absorption spectra of Ag_2S-PEG and Ag_2S-DDT NCs. Fourier Transform Infrared (FTIR) spectra for DDT capped Ag_2S NCs in red and after PEG ligand exchange in blue showing the infrared absorption lines for specific vibrational modes of the two types of ligands.

Characterization and optical spectroscopy

Transmission electron micrographs were recorded on a Talos L120C from Thermo Fischer Scientific. The grids consisted of a Cu mesh covered with a carbon-coated polymer film. Optical absorption measurements were performed with a PerkinElmer Lambda 950 UV-Vis/IR spectrometer in a cuvette with a 1 cm pathlength. The stock samples were diluted ~30 times. Photoluminescence measurements were carried out on an Edinburgh Instruments FS920 spectrometer equipped with a 450 W Xe lamp or an OBIS 520 nm LX 40 mW laser as the excitation source. The emitted light was spectrally dispersed in a 0.22 m monochromator with a liquid nitrogen cooled R5509 photon multiplier tube. The time-resolved measurements were performed using a pulsed OBIS 520 nm LX 40 mW laser as the excitation source and the liquid nitrogen cooled R5509 photon multiplier tube as the detector. For the measurements down to 4 K, an Oxford Instruments liquid He cryostat equipped with a quartz liquid cell sample holder was used. The same excitation sources and detectors as mentioned above were used in addition to an Andor Kymera 328i IR CCD detector for recording NIR emission spectra.

QY measurements

A calibrated spectrofluorometer (Edinburgh Instruments, FLS920) was used for spectral measurements, equipped with a 102 mm inner diameter integrating sphere (Jobin-Yvon). For detection a liquid nitrogen cooled NIR photomultiplier tube (Hamamatsu, R5509-72) was used. For the excitation a CW 808 nm laser from Roithner Lasertechnik GmbH was employed. Temperature controlling and sensing capabilities were incorporated into the integrating sphere using a

temperature controlling chip (W1209), heating element, sample holder thermistor, and sample thermistor.

Section S2. Absorption for Ag_2S-DDT and Ag_2S-PEG NCs

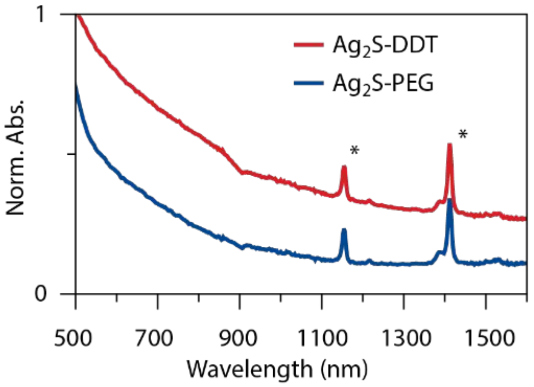

Figure S2 | Absorption spectra of Ag_2S-PEG and Ag_2S-DDT NCs. (**a**) Absorption spectra of both samples of Ag_2S NCs dissolved in tetrachloroethylene with small amounts of $CHCl_3$ left in the solution. The asterisks indicate overtone C-H absorptions originating from the $CHCl_3$.

Section S3. Photoluminescence quantum yield measurements on Ag_2S NCs

The photoluminescence quantum yield (PLQY) is defined by equation S.1 [2]:

$$\text{PLQY} = \frac{\#\,\text{photons emitted}}{\#\,\text{photons absorbed}} = \frac{L_{\text{sample}}}{E_{\text{reference}} - E_{\text{sample}}} \qquad \text{S.1}$$

L_{sample} = Emission intensity

$E_{reference}$ = Intensity of excitation light for a non-absorbing by reference

E_{sample} = Intensity of excitation light not absorbed by the sample

Three measurements for emission and scattering were taken for a quartz cuvette (10 mm) inside of the integrating sphere and the average is reported. As reference, an equivalent amount of the pure solvent was used. The standard deviation was used for the reported errors. The experimental parameters and results of the PLQY measurements are detailed below in tables S1 and S2, respectively.

Table S1 | Experimental parameters for PLQY measurements.

**Ex λ (nm)**	**Excitation grating (nm)**	**Δλ ExBW (nm)**	**Detector**	**Detection grating**	**Δλ EmBW (nm)**	**Filter emission**	**Step (nm)**	**Dwell time (s)**	**λ range (nm)**
808	1200	18	NIR	1200	15	No	2	1	1000-1500
						4 OD	1		790-830
***laser power was set to 100 mW**									
***$CHCl_3$ was used as a reference sample for absorption measurements**									
*** Neutral density filter OD 4 was used for measurements across the excitation range. The filter attenuation coefficient @808nm was determined to be 499.**									

Table S2 | PLQY values for Ag_2S-DDT and Ag_2S-PEG NCs at room temperature.

**Sample**	**Temperature (°C)**	**PLQY (%)**
**Ag_2S-DDT**	24.1	5.1 ± 0.7
**Ag_2S-PEG**	23.8	1.6 ± 0.2

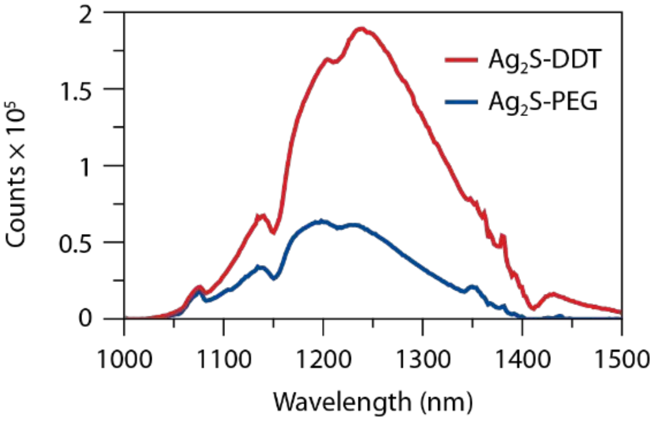

Figure S3 | Emission spectra of the Ag_2S-DDT and Ag_2S-PEG NCs based on which the PLQY values are determined. These are recorded using the setting described in table S1

Section S4. Time-resolved spectroscopy and fitting of the decaycurves

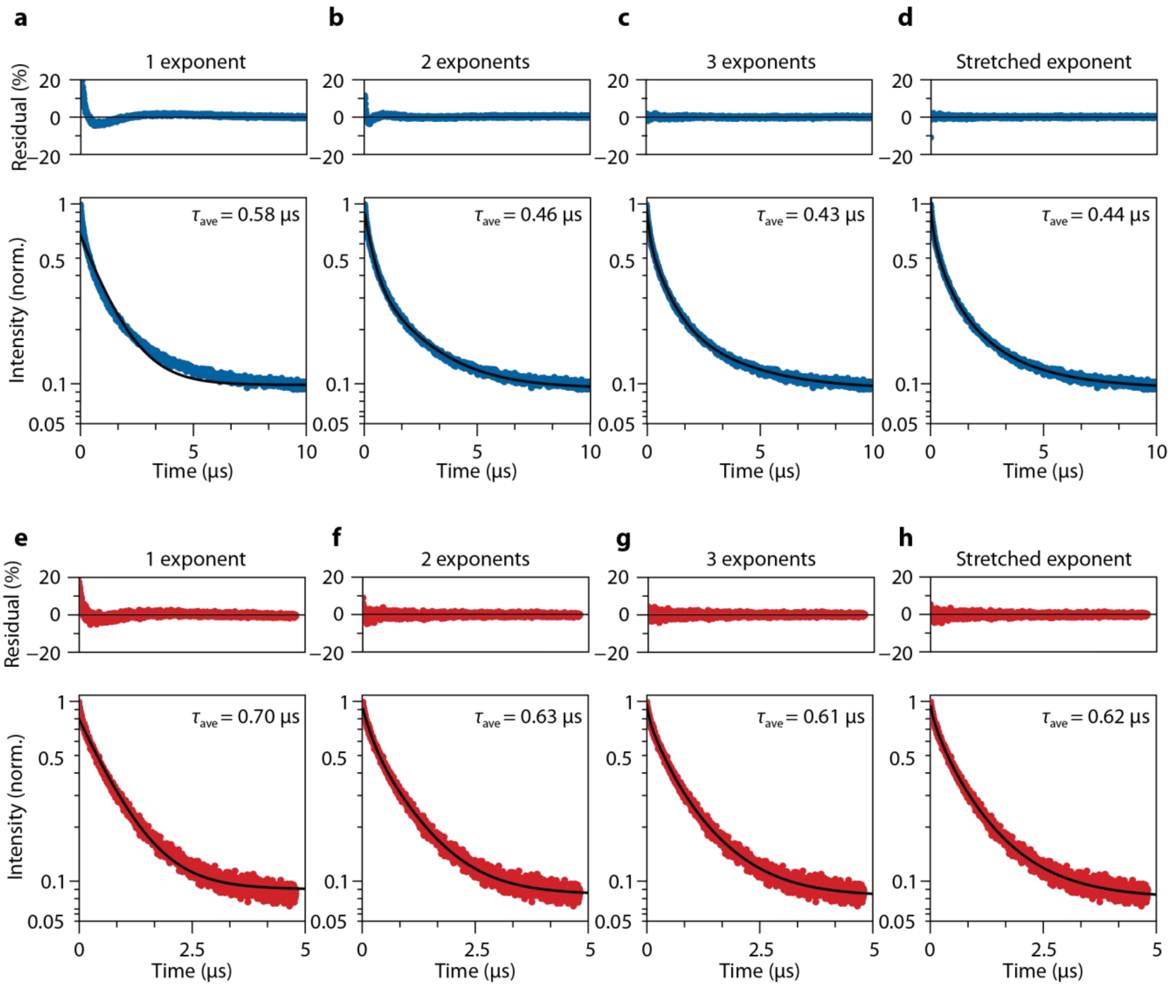

Figure S4 |Room temperature decay curve of Ag_2S (blue) PEG and AG_2S-DDT (red) fitted with different functions and fit residuals, same data as in figure 1d of the main text. (**a**,**e**) A single exponential function of the form $I(t) = A\,e^{-\frac{t}{\tau}} + c$ was fitted to the decay curves and shows a significantly longer lifetime and large fit residual values. (**b**,**f**) The same function as for panels a and d, with one exponent extra, was used to fit the data, based on which an average lifetime was calculated with equation 1 in the main text. There are still some deviations visible in the residual plot between the experimental data and the model fit. The two-exponential fit is used for the data analysis in the main text. (**c,g**) A fit with three exponents or a stretched exponential $I(t) = A\,e^{-(\frac{t}{\tau})^k} + c$ reduces the fit residuals and slightly changes the average lifetime. For the temperature-dependent lifetime data shown in **figure 2b,c** and **figure 3** of the main text we have also used the two-exponential fit procedure. In **section S6** we show the difference between a two- and three-exponential fit procedure in terms of the calculated average lifetime.

Section S5. Temperature-dependent emission spectra of Ag_2S NCs

Figures S5a and **S6a** show the temperature-dependent normalized emission spectra of Ag_2S-PEG NCs and Ag_2S-DDT NCs. Based on these emission spectra, we determined the peak position and full width at half maximum (fwhm) for the Ag_2S-PEG NCs (**figure S5b**) and Ag_2S-DDT NCs (**figure S6b**).

To obtain more insight in the electron-phonon coupling strength for the trapped exciton emission in Ag_2S NCs, we theoretically analyse the temperature behavior of the emission line width (fwhm) and the peak position and compare these results to other semiconductor materials. The emission band broadening has been described by the following equation [3, 4]:

$$\Gamma(T) = \Gamma_0 + \Gamma_{LO}\left[exp\left(\frac{E_{LO}}{k_B T}\right) - 1\right]^{-1}, \quad \text{(S.2)}$$

where Γ_0 represents the bandwidth at 0 K and the second term represents a temperature-dependent homogeneous factor arising from longitudinal optical (LO) electron-phonon interaction, with Γ_{LO} being the corresponding coupling coefficient and E_{LO} the LO phonon energy. Broadening induced by acoustic phonon interaction, given by a term $\Gamma_{ac} = \sigma$ T in the total line width, has been discarded because of the smaller contribution and weak temperature dependence.

By fitting the experimental data to Equation S.2 (see solid line in Figures S5b and S6b), we obtained the parameters listed in Table S3. Similar values (considering the errors) for the electron-phonon interaction parameters were found for both Ag_2S-DDT and Ag_2S-PEG NCs.

The emission peak position has been fitted using a modified Varshni equation [3, 4]:

$$E_g(T) = E_{g0} - 2\,S\,\langle\hbar\omega\rangle\left[exp\left(\frac{\langle\hbar\omega\rangle}{k_B T}\right) - 1\right]^{-1}, \quad \text{(S.3)}$$

where E_{g0} represents the band gap energy of the Ag_2S NCs at 0 K, the parameter *S* is the Huang-Rhys factor, which accounts for the strength of the electron-phonon coupling, and $\langle\hbar\omega\rangle$ represents the average phonon energy. The fitting results are shown in Table S3 (see red solid line in Figures S5b and S6b). Both samples exhibit the same electron-phonon coupling value (*S*) and average phonon energy ($\langle\hbar\omega\rangle$). The *S* value, much larger than 1, indicates a strong electron-phonon coupling. The obtained band gap agrees with that of bulk Ag_2S.

Table S3 | Fitting parameters for the temperature dependence of emission broadening (using Equation S.2) and peak position (using Equation S.3).

Sample	Γ_0 (meV)	γ_{LO} (meV)	E_{LO} (meV)	E_{g0} (eV)	*S*	$\langle\hbar\omega\rangle$ (meV)
Ag_2S-DDT NCs	110 ± 1	88 ± 23	27 ± 4	1.133 ± 0.001	7.9 ± 0.7	55 ± 3

Ag_2S-PEG NCs	96 ± 1	68 ± 16	21 ± 3	1.139 ± 0.001	7.7 ± 0.6	51 ± 3

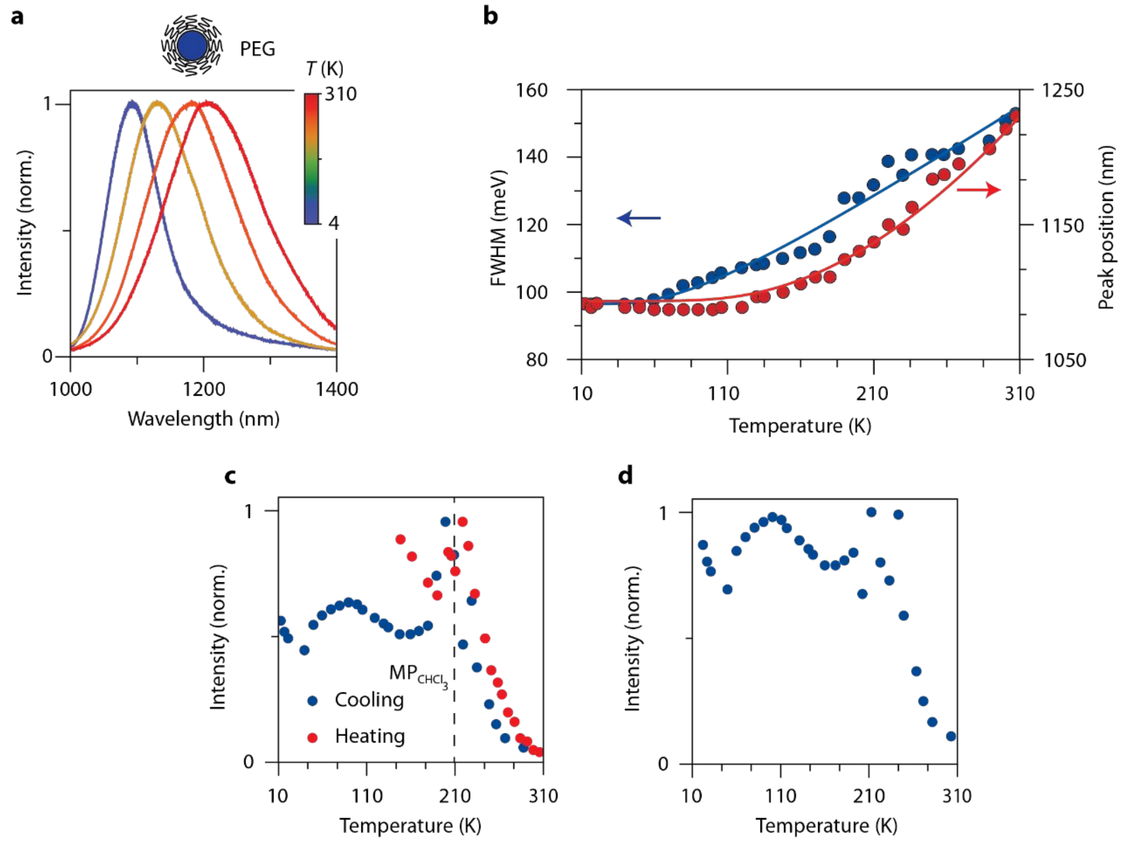

Figure S5 | Temperature-dependent emission spectroscopy on of Ag_2S-PEG NCs. (**a**) Emission spectra of Ag_2S-PEG NCs recorded at (blue to red) 13, 128, 220 and 289 K. (**b**) Plot of the fwhm and the peak position as a function of temperature for the Ag_2S-PEG NCs. The blue line is a fit with equation S.2 to the temperature vs. fwhm data. The red line is a fit of equation S.3 to the temperature vs. peak position data. Fit outcomes can be found in table S3 (**c**) Integrated photoluminescence intensity plotted as a function of temperature recorded while cooling down (blue) and while warming up (red). The dashed line marks the melting temperature of $CHCl_3$. (**d**) Same as in panel **c** but corrected for the intensity jump created by the solidification of $CHCl_3$.

The integrated emission intensities as function of temperature for Ag_2S-PEG NCs are shown in **figure S5c** and **figure S6c**. Around 210 K, a discontinuity is observed in the integrated intensity for both samples. This temperature coincides with the melting point of the $CHCl_3$ in which the NCs are dispersed (dashed black line). To verify the intensity jump was caused by a phase transition induced change in the collection efficiency, we also recorded spectra while warming up. During warming up, the intensity jump takes place at a slightly different set temperature, indicating that a (delayed) phase transition is indeed responsible for the jump in emission intensity. To correct the data, we took the average intensity of the datapoints in the temperature range 13 to 200 K and subtracted that from all datapoints above 210 K (**figure S5d, figure S6d**). The drop in intensity upon solidification of the chloroform solvent is not because of quenching but because of a change in optically active volume probed. In a liquid the light can fully penetrate and probe a large volume of solvent with luminescing nanocrystals. Upon solidification, the light path changes and due to strong scattering only probes a surface layer. It can be compared to measuring a transparent single crystal vs. a microcrystalline powder. Also there, the emission intensity collected from a single crystal is

larger than for microcrystalline powder. How much the difference is, depends on the alignment of the sample and may vary.

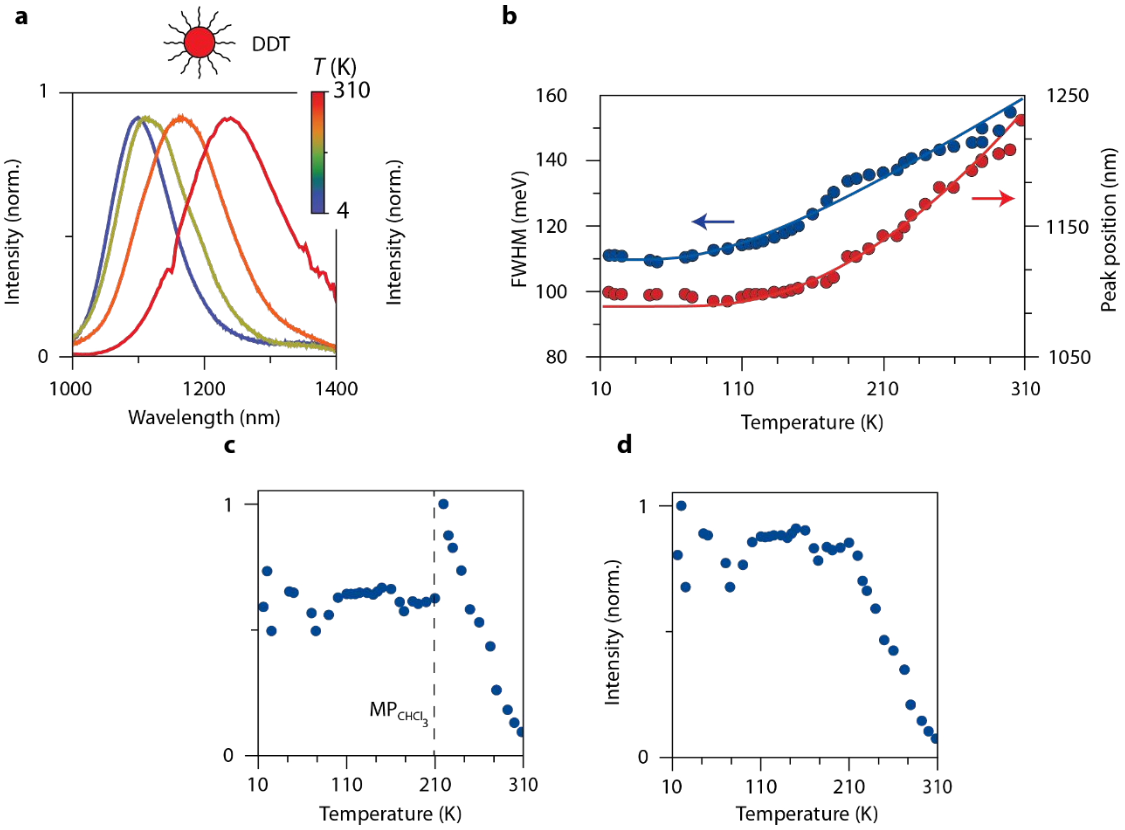

Figure S6 | Temperature-dependent emission spectroscopy of Ag_2S-DDT NCs. (**a**) Emission spectra of Ag_2S-DDT NCs recorded at (blue to red) 13, 128, 220 and 289 K. (**b**) Plot of the fwhm and the peak position as a function of temperature for the Ag_2S-DDT NCs. The blue line is a fit with equation S.2 to the temperature vs. fwhm data. The red line is a fit of equation S.3 to the temperature vs. peak position data. Fit outcomes can be found in table S3 (**c**) Integrated photoluminescence intensity plotted as a function of temperature recorded while cooling down (blue). The dashed line marks the melting temperature of $CHCl_3$. (**d**) Same as in panel **c** but corrected for the intensity jump created by the solidification of $CHCl_3$.

Section S6. Temperature-dependent decay measurements

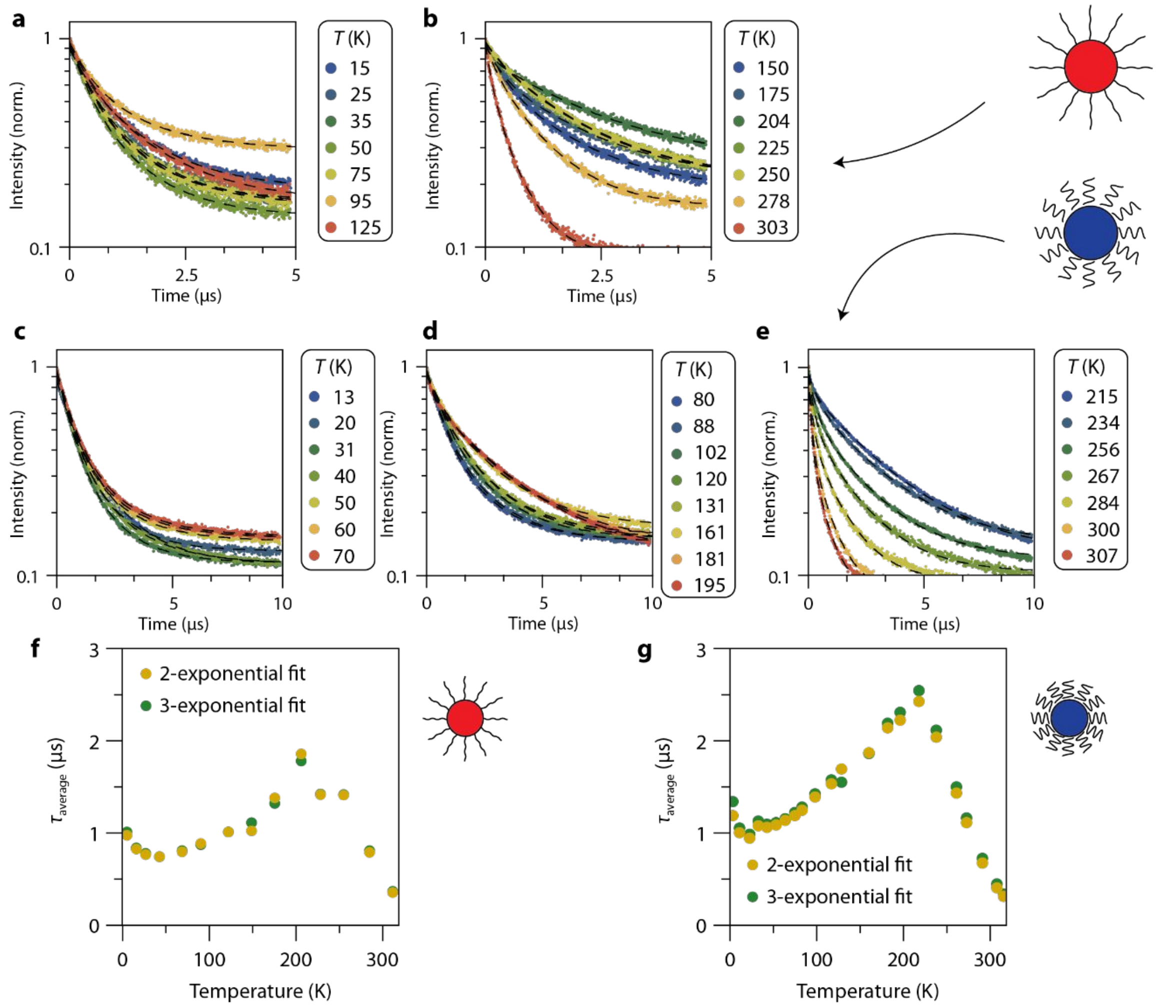

Figure S7 | Overview of temperature-dependent time-resolved emission measurements for the Ag_2S-PEG and Ag_2S-DDT NCs. (**a**) Temperature-dependent decay curves of Ag_2S-DDT NCs between 15 and 125 K including fits. (**b**) Same as in **a**, but between the 150 and 303 K. (**c**) Temperature-dependent decay curves of Ag_2S-PEG NCs between 15 and 70 K including fits. Same is in **c**, but between the 80 and 195 K. (**d**) Same is in **c**, but between the 215 and 307 K. Plot of average lifetime versus temperature based on a two-exponential fit and a three exponential fit procedure for (**f**) Ag_2S-DDT and (**g**) Ag_2S-PEG NCs. It illustrates that there are very small deviations between the fit procedures but these do not affect the observed temperature dependence of the average luminescence decay time.

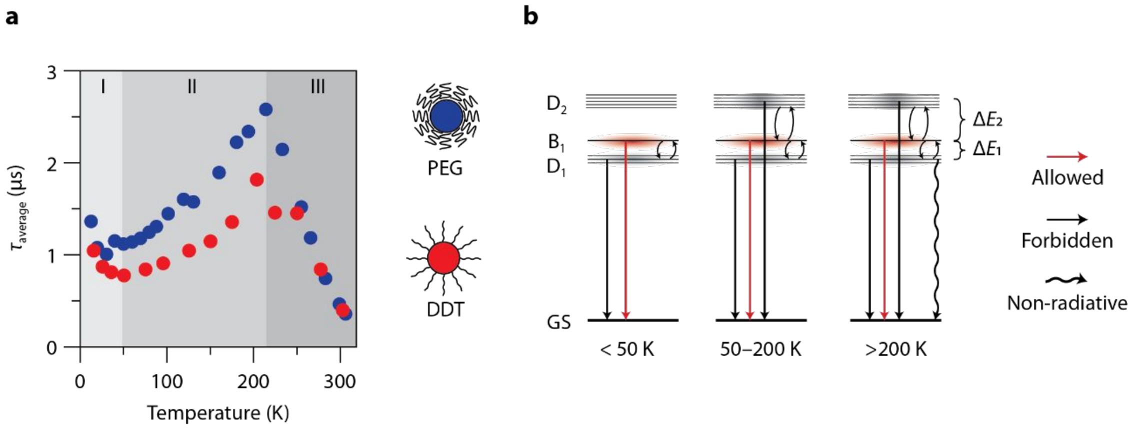

Figure S8 | Temperature-dependent luminescence lifetime of Ag_2S NCs and the proposed three-level excited state model for Ag_2S. (**a**) Plot of the average lifetime as a function of temperature for the Ag_2S-PEG NCs (blue) and the Ag_2S-DDT NCs (red). At 0 K only the lowest dark state (D_1) is populated and all emission takes place from there. With increasing temperature, the lifetime becomes shorter because increasingly, the bright B_1 excited state is thermally populated. Upon increasing the temperature above 50 K (region II), the higher energy excited dark state D_2 is populated which lengthens the average observed lifetime. In region III non-radiative processes increasingly dominate the observed decay time and quench the emission. (**b**) Same plot as in **figure 4** in the main text showing the temperatures at which the different states become thermally occupied and corresponding to the onset of Regions I, II and III in (**a**).

References

[1] I. Z. Gutierrez *et al.*, “Boosting the Near-Infrared Emission of Ag_2S Nanoparticles by a Controllable Surface Treatment for Bioimaging Applications,” *ACS Appl. Mater. Interfaces*, vol. 14, no. 4, pp. 4871–4881, 2022, DOI: 10.1021/acsami.1c19344.

[2] C. M. S. Jones *et al.*, “Effect of light scattering on upconversion photoluminescence quantum yield in microscale-to-nanoscale materials,” *Opt. Express*, vol. 28, no. 15, p. 22803, 2020, DOI: 10.1364/oe.398353.

[3] C. Ji et al., "Temperature dependent photoluminescence of Ag_2Se Quantum Dots", *J. Phys. Chem. C* vol. 119, pp. 13841−13846, 2015, DOI: 10.1021/acs.jpcc.5b01030

[4] J.A. Steele et al., "Giant electron-phonon coupling and deep conduction band resonance in metal halide double perovskite", *ACS Nano* vol. 12, pp. 8081-8090, 2018, DOI: 10.1021/acsnano.8b02936
