# Supplementary material for: New Insights in Luminescence and Quenching Mechanisms of Ag2S Nanocrystals through Temperature-Dependent Spectroscopy
Source: J Phys Chem Lett. 2024 Aug 8;15(33):8420–6. doi: 10.1021/acs.jpclett.4c01439 (PMC11345845; doi:10.1021/acs.jpclett.4c01439)
Supplement: Supplementary file 2 — jz4c01439_si_002.pdf [file jz4c01439_si_002.pdf]

jz-2024-01439q.R1

Name: Peer Review Information for "New Insights in Luminescence and Quenching Mechanisms of Ag₂S Nanocrystals through Temperature-Dependent Spectroscopy"

First Round of Reviewer Comments

Reviewer: 1

Comments to the Author

In this manuscript, the authors investigate the luminescence and quenching mechanisms of Ag2S nanocrystals through temperature-dependent emission and lifetime measurements. The authors find unusual decay behavior variations at low temperatures and elucidate this phenomenon using a model with multiple thermally coupled excited states. This would be enlightening to thermal quenching mechanism of Ag2S nanocrystals. I am generally supportive of publication for this work, but I also have identified areas in which I believe changes will improve the work.

Comments:

1. The authors take DDT and PEG capped Ag2S nanocrystals as the object of study, yet fail to provide a thorough explanation for their selection. In addition, the experimental results for nanocrystals capped with two ligands were quite different, and the authors should explain the effect of the distinct ligands.

2. Why does the solidification of the solvent chloroform cause such a large change in the integrated emission intensity?

3. The authors attribute the fluctuations in emission intensity below 200 K to external factors. Do these factors have the same effect on the results of the temperature-dependent luminescence lifetime, especially below 200 K? The authors should clarify this issue.

4. The meaning of some capitals and symbols is not clear in Figure 4. Specific meanings of capitals and symbols need to be explained in the text or in the figure legends.

5. The temperature dependent PL have also be performed on other Cd-free QDs (Nano Lett. 2023, 23, 9, 4032–4038), such as ZnSe, ZnSeTe (Appl. Phys. Rev. 11, 021419 (2024)), these results may provide clues for Ag2Se QDs.

6. The authors need to pay attention to the issue of subscripts in “Ag2S”, for example the phrase “Ligand exchange to Ag2S-PEG NCs” in Section S1.

Reviewer: 2

Comments to the Author

The manuscript investigated the luminescence and quenching mechanism of Ag2S NCs capped with either DDT or SH-PEG using temperature-dependent spectroscopy. From 200 to 300 K, both emission and lifetime measurements reveal similar and strong thermal quenching, indicating an intrinsic quenching process. Below 200 K, the emission intensity remains constant while the lifetime shortens until 50 K, followed by a lengthening of the lifetime upon further cooling to 10 K. These results were explained in terms of multiple thermally coupled excited states, with a lowest energy dark state, a higher bright state followed by dark states at even higher energies. This work offers valuable insights for understanding the optical properties of Ag2S NCs and the thermal quenching mechanism underlying their temperature sensing capabilities. This work make sense and requires a major revision before publication.

1. The ligand exchange reaction is influenced by many factors including the temperature, NCs' surface structure and differences in ligand binding forces. Since both are monosulfhydryl ligands, what are the factors driving SH-PEG replacement of DDT? Is this process efficient?

2. Please provide more data to verify the successful ligand exchange, like FTIR.

3. In Figure 1c, the emission peak position presented a blue-shift after ligand exchange. Please explain this phenomenon in more detail.

4. In SI Section S3, the QY measurement was detailed described. But the original test spectra were missing, please revise them.

5. Regarding the statement "Decay curves were fitted with a two-exponential function which results in an average lifetime", there is no discussion about the two lifetimes components such as the value and the meaning.

6. From the temperature-dependent emission spectra, more information about radiative and nonradiative relaxation processes could be obtained, as well as exciton-phonon coupling. Please analyze the data more thoroughly.

7. The measured Ag2S NCs underwent sonication procedure in CHCl3 which may resulting in chlorine-passivation. Whether the data and conclusions for Ag2S NCs obtained using the passivated NC are persuasive? In addition, the conclusion for "perfectly passivated NCs" may be inappropriate. Will other better Cl-passivated Ag2S NCs with higher PLQY (Adv. Optical Mater. 2022, 10, 2102806) lead to different conclusions?

Author's Response to Peer Review Comments:

Response Letter to Reviewers for de Wit *et al*. (jz-2024-01439q)

We thank the reviewers for their insightful comments, which helped us improve the manuscript. Below we address the comments of the reviewers by repeating the comment in blue, followed by our response and actions in black. Adjustments of the manuscript are shown in red.

Reviewer1: **1**

Recommendation: This paper may be publishable, but major revision is needed; I would like to be invited to review any future revision.

Comments:
In this manuscript, the authors investigate the luminescence and quenching mechanisms of Ag2S nanocrystals through temperature-dependent emission and lifetime measurements. The authors find unusual decay behavior variations at low temperatures and elucidate this phenomenon using a model with multiple thermally coupled excited states. This would be enlightening to thermal quenching mechanism of Ag2S nanocrystals. I am generally supportive of publication for this work, but I also have identified areas in which I believe changes will improve the work.

Our response: We thank the reviewer for the positive evaluation and constructive feedback. Below we will address all remarks point by point.

1. The authors take DDT and PEG capped Ag2S nanocrystals as the object of study, yet fail to provide a thorough explanation for their selection. In addition, the experimental results for nanocrystals capped with two ligands were quite different, and the authors should explain the effect of the distinct ligands.

Our response: The motivation for the choice of ligands is indeed not clearly discussed in the manuscript. DDT is widely used as capping molecule in this type of efficiently luminescing nanocrystals. The hydrophobic nature makes DDT capped nanocrystals less suitable for bioapplications and ligand exchange by PEG is commonly used to render the nanocrystals biocompatible. In the revised manuscript we have included this information. In addition, also in response to the comment by reviewer 2, we have included FTIR spectra to demonstrate the successful ligand exchange. The trends we observe for the temperature dependent emission intensity and luminescence lifetime are the same for both types of nanocrystals which provides evidence that the observed trends are intrinsic to the Ag_2S NCs and not related to their surface chemistry.

Changes in the manuscript: The ligand exchange procedure is now described in in the supporting information along with an FTIR spectrum before and after ligand exchange:

Ligand exchange to Ag_2S-PEG NCs

For the ligand exchange, 2 mg of PEG was added to 1 mg of sonicated Ag_2S NCs dispersed in 1 ml $CHCl_3$. The reaction was stirred vigorously for 30 min after which hexane was added to destabilize the dispersion. The precipitate was collected and redispersed in $CHCl_3$. The incorporation of the PEG-SH molecules on the surface of the nanoparticles is driven by the sonochemical treatment

carried out prior to the incorporation of the PEG molecules. This process produces an etching reaction on the surface of the nanoparticles that reduces the number of DDT molecules anchored on the surface and renders NPs with pristine regions that can be functionalized with the PEG moieties. A complete description of the process can be found in Ref. [1]. In addition, to confirm the success of the PEG functionalization Figure S1 shows FTIR spectra of DDT and PEG functionalized nanoparticles. Here, we can observe that the incorporation of the PEG-SH modifies the FTIR spectrum of the nanoparticles, exhibiting bands that are specific of PEG molecules like the C-O stretching vibration at 1091 cm^{-1} and that does not appear in DDT functionalized nanoparticles. For the PEG-capped NCs we observe the CH_2 stretching vibrations at 2843 cm^{-1}. In addition, bands at 1478 and 1442 cm^{-1} can be assigned to the bending mode of the C-H group, while the bands that appeared at 1279 and 1229 cm^{-1} could be attributed to the C-H twisting vibrations. By contrast, the DDT functionalized nanoparticles exhibits a completely different FTIR spectrum that features sharp bands at 2922 and 2852 cm^{-1}, which can be assigned to the asymmetric methyl stretching and asymmetric/symmetric methylene stretching modes respectively. In summary, the FTIR spectra demonstrate clearly the incorporation of the PEG moieties and the success of the nanoparticle functionalization.

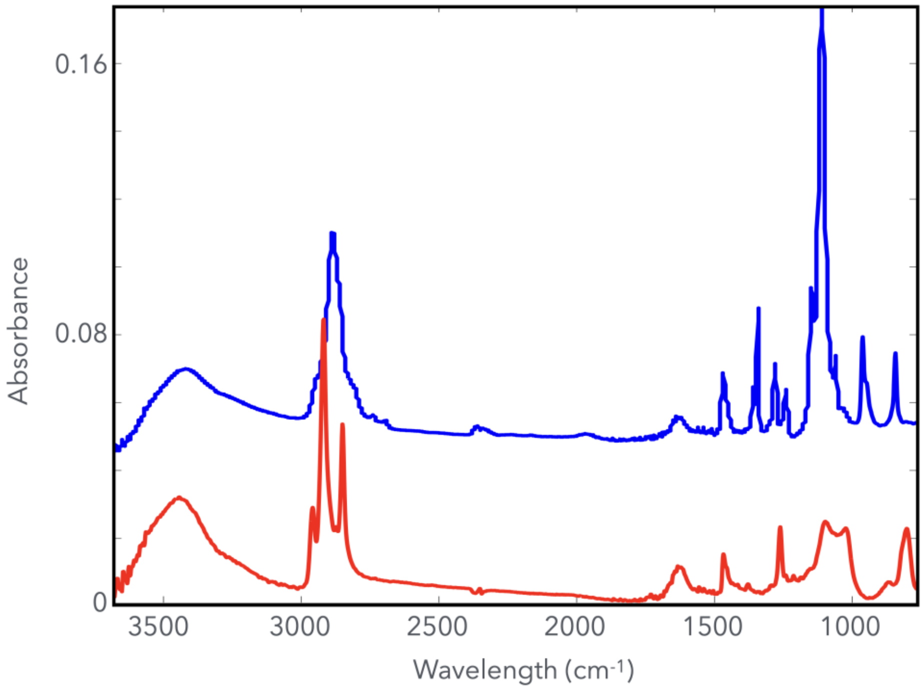

Figure S1 | FTIR absorption spectra of Ag_2S-PEG and Ag_2S-DDT NCs. Fourier Transform Infrared spectra for DDT capped Ag_2S NCs in red and after PEG ligand exchange in blue showing the infrared absorption lines for specific vibrational modes of the two types of ligands.

In the main text we now refer to capping exchange procedure and the FTIR analysis done to confirm the successful ligand exchange:

On page 4 of the manuscripts we added: Experimental details can be found in Supporting Information Section S1 describing the ligand exchange procedure and showing FTIR spectra before and after ligand exchange (**figure S1**) confirming successful exchange.

2. Why does the solidification of the solvent chloroform cause such a large change in the integrated emission intensity?

Our response: The drop in intensity upon solidification is close to 40% with respect to the liquid chloroform. This is indeed a large drop, but this is often observed when a transparent solution solidifies to become an opaque scattering medium. In a liquid the light can fully penetrate and probe a large volume of solvent with luminescing nanocrystals. Upon solidification, the light path changes and due to strong scattering only probes a surface layer. This typically causes a drop in emission intensity, not because of quenching but because of the change in optically active volume probed. It can be compared to measuring on a transparent single crystal and a microcrystalline powder. Also there, the emission intensity collected from a single crystal is larger than for microcrystalline powder. How much the difference is, depends on the alignment of the sample and may vary. In the revised manuscript this now explained in the supporting information.

Changes in the manuscript: In the text concerning Figure S6 we have added: 'The drop in intensity upon solidification of the chloroform solvent is not because of quenching but because of the change in optically active volume probed. In a liquid the light can fully penetrate and probe a large volume of solvent with luminescing nanocrystals. Upon solidification, the light path changes and due to strong scattering only probes a surface layer. It can be compared to measuring a transparent single crystal vs. a microcrystalline powder. Also there, the emission intensity collected from a single crystal is larger than for microcrystalline powder. How much the difference is, depends on the alignment of the sample and may vary.'

3. The authors attribute the fluctuations in emission intensity below 200 K to external factors. Do these factors have the same effect on the results of the temperature-dependent luminescence lifetime, especially below 200 K? The authors should clarify this issue.

Our response: The factors we mention that can explain the fluctuations in emission intensity but do not affect the shape of the photoluminescence decay curves. Luminescence decay time is considered an inherent property of luminescent species, unaffected by external experimental factors, such as the intensity of the excitation pulse (provided we do not saturate the detector – we are far from this regime in our experiments) and the efficiency for luminescence collection. Therefore, fluctuations in intensity are not reflected in the decay times. During a decay measurement the time dependent emission intensity is summed over many excitation pulses and the resulting decay luminescence curve represents the decay kinetics of the emitters in the luminescent material.

Changes in the manuscript: In the text under Figure 2 we added to the discussion on intensity variations 'Note that variations in intensity due to artefacts discussed above do not affect the temperature dependent luminescence lifetime as the decay kinetics are an inherent property of luminescent species and the dynamics are unaffected by external factors that cause changes in integrated emission intensity. '

4. The meaning of some capitals and symbols is not clear in Figure 4. Specific meanings of capitals and symbols need to be explained in the text or in the figure legends.

Our response: The referee is correct to note that this is not clear. We have included the meaning of the symbols in the revised version so that the meaning is clear and helps to better understand figure 4.

D1 → dark state 1 B1 → bright state

D2 → dark state 2 GS → ground state

Changes in the manuscript: Figure 4 in the main manuscript has been replaced by a new Figure 4 with clear figure legends.

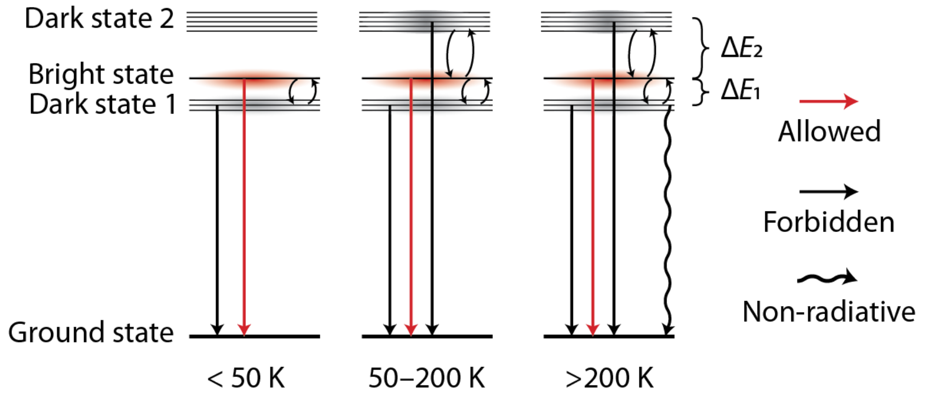

5. The temperature dependent PL have also be performed on other Cd-free QDs (Nano Lett. 2023, 23, 9, 4032–4038), such as ZnSe, ZnSeTe (Appl. Phys. Rev. 11, 021419 (2024)), these results may provide clues for Ag2Se QDs.

Our response: We recognize the relevance of these studies that shed light on the photophysical mechanisms in semiconductor nanocrystals that are environmentally friendly alternatives to Pb and Cd containing ones. The suggested manuscripts are nice pieces of work, yet they study a different material (ZnSe-based QDs), focus on the investigation of optical gain in quantum dots and no temperature-dependent measurements are reported. Because of the above, we feel the publications have less relevance in the context of the our investigation.

6. The authors need to pay attention to the issue of subscripts in “Ag2S”, for example the phrase “Ligand exchange to Ag2S-PEG NCs” in Section S1.

Our response: We thank the reviewer for noting these inconsistencies. We have rechecked the manuscript and SI and changed the notation if necessary.

Changes in the manuscript: Subscripts have checked and corrected where needed.

Additional Questions:
Urgency: **High**
Significance: **High**
Novelty: **High**
Scholarly Presentation: **High**
Is the paper likely to interest a substantial number of physical chemists, not just specialists working in the authors' area of research?: **Yes**

Reviewer2:

Recommendation: This paper may be publishable, but major revision is needed; I would like to be invited to review any future revision.

Comments:
The manuscript investigated the luminescence and quenching mechanism of Ag2S NCs capped with either DDT or SH-PEG using temperature-dependent spectroscopy. From 200 to 300 K, both emission and lifetime measurements reveal similar and strong thermal quenching, indicating an intrinsic quenching process. Below 200 K, the emission intensity remains constant while the lifetime shortens until 50 K, followed by a lengthening of the lifetime upon further cooling to 10 K. These results were explained in terms of multiple thermally coupled excited states, with a lowest energy dark state, a higher bright state followed by dark states at even higher energies. This work offers valuable insights for understanding the optical properties of Ag2S NCs and the thermal quenching mechanism underlying their temperature sensing capabilities. This work make sense and requires a major revision before publication.

Our response: We appreciate the positive evaluation of our manuscript by the reviewer and valuable comments which have helped to improve the manuscript.

1. The ligand exchange reaction is influenced by many factors including the temperature, NCs' surface structure and differences in ligand binding forces. Since both are monosulfhydryl ligands, what are the factors driving SH-PEG replacement of DDT? Is this process efficient?

Our response: This is a valid point that was also raised by Reviewer1. Ligand exchange procedures for these nanocrystals have been described in earlier work by some of the authors. However, it is good to include this information as it is important to understand how the ligand exchange was done and also provide evidence that the ligand exchange was successful. In the revised manuscript we include a description of the procedure and also FTIR spectra to show that the ligand exchange was successful.

Changes in the manuscript: The ligand exchange procedure is now described in in the supporting information along with an FTIR spectrum before and after ligand exchange:

Ligand exchange to Ag_2S-PEG NCs

For the ligand exchange, 2 mg of PEG was added to 1 mg of sonicated Ag_2S NCs dispersed in 1 ml $CHCl_3$. The reaction was stirred vigorously for 30 min after which hexane was added to destabilize the dispersion. The precipitate was collected and redispersed in $CHCl_3$. The incorporation of the PEG-SH molecules on the surface of the nanoparticles is driven by the sonochemical treatment carried out prior to the incorporation of the PEG molecules. This process produces an etching reaction on the surface of the nanoparticles that reduces the number of DDT molecules anchored on the surface and renders NPs with pristine regions that can be functionalized with the PEG moieties. A complete description of the process can be found in Ref. [1]. In addition, to confirm the success of the PEG functionalization Figure S1 shows FTIR spectra of DDT and PEG functionalized nanoparticles. Here, we can observe that the incorporation of the PEG-SH modifies the FTIR spectrum of the nanoparticles, exhibiting bands that are specific of PEG molecules like the C-O stretching vibration at 1091 cm^{-1} and that does not appear in DDT functionalized nanoparticles. For the PEG-capped NCs we observe the CH_2 stretching vibrations

at 2843 cm^{-1}. In addition, bands at 1478 and 1442 cm^{-1} can be assigned to the bending mode of the C-H group, while the bands that appeared at 1279 and 1229 cm^{-1} could be attributed to the C-H twisting vibrations. By contrast, the DDT functionalized nanoparticles exhibits a completely different FTIR spectrum that features sharp bands at 2922 and 2852 cm^{-1}, which can be assigned to the asymmetric methyl stretching and asymmetric/symmetric methylene stretching modes respectively. In summary, the FTIR spectra demonstrate clearly the incorporation of the PEG moieties and the success of the nanoparticle functionalization.

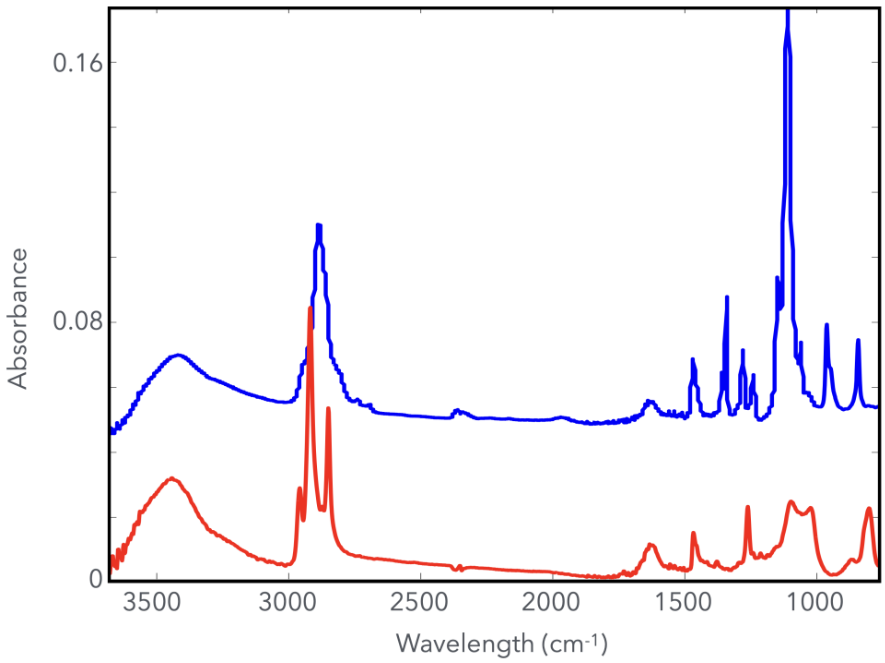

Figure S1 | FTIR absorption spectra of Ag_2S-PEG and Ag_2S-DDT NCs. Fourier Transform Infrared spectra for DDT capped Ag_2S NCs in red and after PEG ligand exchange in blue showing the infrared absorption lines for specific vibrational modes of the two types of ligands.

In the main text we now refer to capping exchange procedure and the FTIR analysis done to confirm the successful ligand exchange:

On page 4 of the manuscripts we added: Experimental details can be found in Supporting Information Section S1 describing the ligand exchange procedure and showing FTIR spectra before and after ligand exchange (**figure S1**) confirming successful exchange.

2. Please provide more data to verify the successful ligand exchange, like FTIR.

Our response: Following the reviewer suggestion and in line with the response above, we have included the FTIR spectra of both types of nanoparticles, see Figure S1. Here one can see that after the ligand exchange reaction is successful as the FTIR spectrum of the PEG functionalized nanoparticles is different from the DDT capped NCs and shows features that are consistent with PEG capping.

Changes in the manuscript: See above

3. In Figure 1c, the emission peak position presented a blue-shift after ligand exchange. Please explain this phenomenon in more detail.

Our response: This has been reported before for various types of chalcogenide nanocrystals. In general, thiol ligands give rise to a slightly redshifted emission. For chalcogenide quantum dots experiencing quantum confinement this is typically explained by a reduction in the confinement energy as a result of some delocalization over the more covalent sulfur ligands. For our Ag_2S NCs the blue shift can be explained as a modification of the electronic-distribution which is related to a difference in the atomistic arrangement on the NP surface that dependent on the capping agent and the environment of the NPs (*ACS Omega* 3 pp. 393–405 (2018), doi.org/10.1021/acsomega.7b01451). As the temperature dependence and quenching of the trapped exciton emission are the focus of this manuscript we feel it will distract from the main message of the manuscript to discuss this aspect.

4. In SI Section S3, the QY measurement was detailed described. But the original test spectra were missing, please revise them.

Our response: We agree it is useful to include these figures and have added them to the SI as Fig. S3. In addition, it is useful to provide a more elaborate description of the quantum yield measurements and the experimental settings used. In the revised manuscript we now include a more informative section on the quantum yield measurements.

Changes in the manuscript: In the Supporting Information the section on quantum yield measurements has been extended:

The photoluminescence quantum yield (PLQY) is defined by equation S.1 [2]:

$$\text{PLQY} = \frac{\#\text{ photons emitted}}{\#\text{ photons absorbed}} = \frac{L_{\text{sample}}}{E_{\text{reference}} - E_{\text{sample}}} \qquad \text{S.1}$$

L_{sample} = Emission intensity

$E_{reference}$ = Intensity of excitation light for a non-absorbing by reference

E_{sample} = Intensity of excitation light not absorbed by the sample

Three measurements for emission and scattering were taken for a quartz cuvette (10 mm) inside of the integrating sphere and the average is reported. As reference, an equivalent amount of the pure solvent was used. The standard deviation was used for the reported errors. The experimental parameters and results of the PLQY measurements are detailed below in tables S1 and S2, respectively.

Table S1 | Experimental parameters for PLQY measurements.

Ex λ (nm)	Excitation grating (nm)	Δλ ExBW (nm)	Detector	Detection grating	Δλ EmBW (nm)	Filter emission	Step (nm)	Dwell time (s)	λ range (nm)

<table>
<tr><td rowspan="2">808</td><td rowspan="2">1200</td><td rowspan="2">18</td><td rowspan="2">NIR</td><td rowspan="2">1200</td><td rowspan="2">15</td><td>No</td><td>2</td><td rowspan="2">1</td><td>1000-1500</td></tr>
<tr><td>4 OD</td><td>1</td><td>790-830</td></tr>
<tr><td colspan="10">*laser power was set to 100 mW</td></tr>
<tr><td colspan="10">*$CHCl_3$ was used as a reference sample for absorption measurements</td></tr>
<tr><td colspan="10">* Neutral density filter OD 4 was used for measurements across the excitation range. The filter attenuation coefficient @808nm was determined to be 499.</td></tr>
</table>

Table S2 | PLQY values for Ag_2S-DDT and Ag_2S-PEG NCs at room temperature.

Sample	Temperature (°C)	PLQY (%)
**Ag_2S-DDT**	24.1	5.1 ± 0.7
**Ag_2S-PEG**	23.8	1.6 ± 0.2

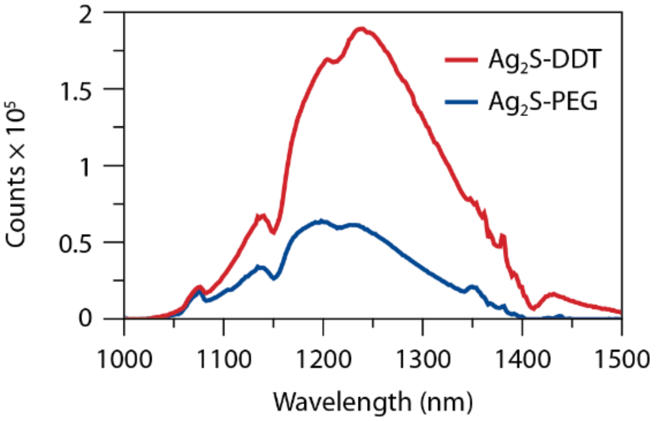

Figure S3 | Emission spectra of the Ag_2S-DDT and Ag_2S-PEG NCs based on which the PLQY values are determined. These are recorded using the setting described in table S1

5. Regarding the statement "Decay curves were fitted with a two-exponential function which results in an average lifetime", there is no discussion about the two lifetimes components such as the value and the meaning.

Our response: The experimentally observed decay curves deviate from a single exponential decay, which is why we decided to use a multiexponential decay function to fit the experimental curves. Differences in non-radiative decay processes related to (surface) defects lead to Ag_2S NCs with different decay rates. We also determined the minimum number of exponential functions required to achieve accurate fitting results and found that two exponentials are sufficient to accurately reproduce the experimental curves. In addition, we tested a stretched exponential and three exponential functions and found that the results were very similar to those obtained with the two-exponential function. The quality of the fits with three-exponential functions or a stretched function is only marginally better, and the trends exactly the same as for the bi-exponential procedure. We have discussed our choice to fit the room temperature

experimental data with a two-exponential function in the main text and SI Section S4. In the manuscript we stress clearly that the bi-exponential fit provides a good fit with the experimental curves but that the actual decay is multi-exponential: ‘A two-exponential function gives a good description of the experimentally observed decay behavior (**see figure S4**) and can be used to quantify changes in decay behavior. However, it is important to realize that decay rates vary for different Ag_2S NCs as a result of many different de-excitation rates originating from variations in surface trap concentrations. The fit to a bi-exponential function should not be understood as there being only two different decay rates for different Ag_2S NCs.’

Changes in the manuscript: In the revised manuscript we added: The two lifetimes provide a good description of the multi-exponential decay kinetics of the ensemble of Ag_2S NCs.

6. From the temperature-dependent emission spectra, more information about radiative and nonradiative relaxation processes could be obtained, as well as exciton-phonon coupling. Please analyze the data more thoroughly.

Our response:

We have theoretically analyzed the temperature behavior of the emission line width and the peak position. From this analysis we have determined the Huang-Rhys parameter S for the electron-phonon coupling and shown that the temperature dependent FWHM follows behavior that is typically observed for emission bands in the strong coupling regime.

Changes in the manuscript: We added the analysis in Section S5 of the Supporting Information:

‘To obtain more insight in the electron-phonon coupling strength for the trapped exciton emission in Ag_2S NCs, we theoretically analyse the temperature behavior of the emission line width (fwhm) and the peak position and compare these results to other semiconductor materials. The emission band broadening has been described by the following equation [3,4]:

$$\Gamma(T) = \Gamma_0 + \Gamma_{LO}\left[exp\left(\frac{E_{LO}}{k_B T}\right) - 1\right]^{-1}, \qquad (S.2)$$

where Γ_0 represents the bandwidth at 0 K and the second term represents a temperature-dependent homogeneous factor arising from longitudinal optical (LO) phonon-exciton interaction, with Γ_{LO} being the corresponding coupling coefficient and E_{LO} the LO phonon energy. Broadening induced by acoustic phonon-exciton interaction, given by the term $\Gamma_{ac} = \sigma T$ in the total line width, has been discarded because of the smaller contribution and weak temperature dependence.

By fitting the experimental data to Equation S.2 (see solid line in Figures S5b and S6b), we obtained the parameters listed in Table S3. Similar values (considering the errors) for the electron-phonon interaction parameters were found for both Ag_2S-DDT and Ag_2S-PEG NCs.
The emission peak position has been reproduced using a modified Varshni equation [3, 4]:

$$E_g(T) = E_{g0} - 2\,S\,\langle\hbar\omega\rangle\left[exp\left(\frac{\langle\hbar\omega\rangle}{k_B T}\right) - 1\right]^{-1}, \qquad (S.3)$$

where E_{g0} represents the band gap energy of the Ag_2S NCs at 0 K, the parameter *S* is the Huang-Rhys factor, which accounts for the strength of the electron-phonon coupling, and $\langle\hbar\omega\rangle$ represents the average phonon energy. The fitting results are shown in Table S3 (see red solid line in Figures S5b and S6b). Both samples exhibit the same electron-phonon coupling value (*S*) and

average phonon energy ($\langle \hbar\omega \rangle$). The S value, much larger than 1, indicates a strong electron-phonon coupling. The obtained band gap agrees with that of bulk Ag_2S.

Table S3 | Fitting parameters for the temperature dependence of emission broadening (using Equation S2) and peak position (using Equation S3).

Sample	Γ_0 (meV)	Γ_{LO} (meV)	E_{LO} (meV)	E_{g0} (eV)	S	$\langle \hbar\omega \rangle$ (meV)
Ag_2S-DDT NCs	110 ± 1	88 ± 23	27 ± 4	1.133 ± 0.001	7.9 ± 0.7	55 ± 3
Ag_2S-PEG NCs	96 ± 1	68 ± 16	21 ± 3	1.139 ± 0.001	7.7 ± 0.6	51 ± 3

In addition, the fits are now shown in Figs S5 and S6:

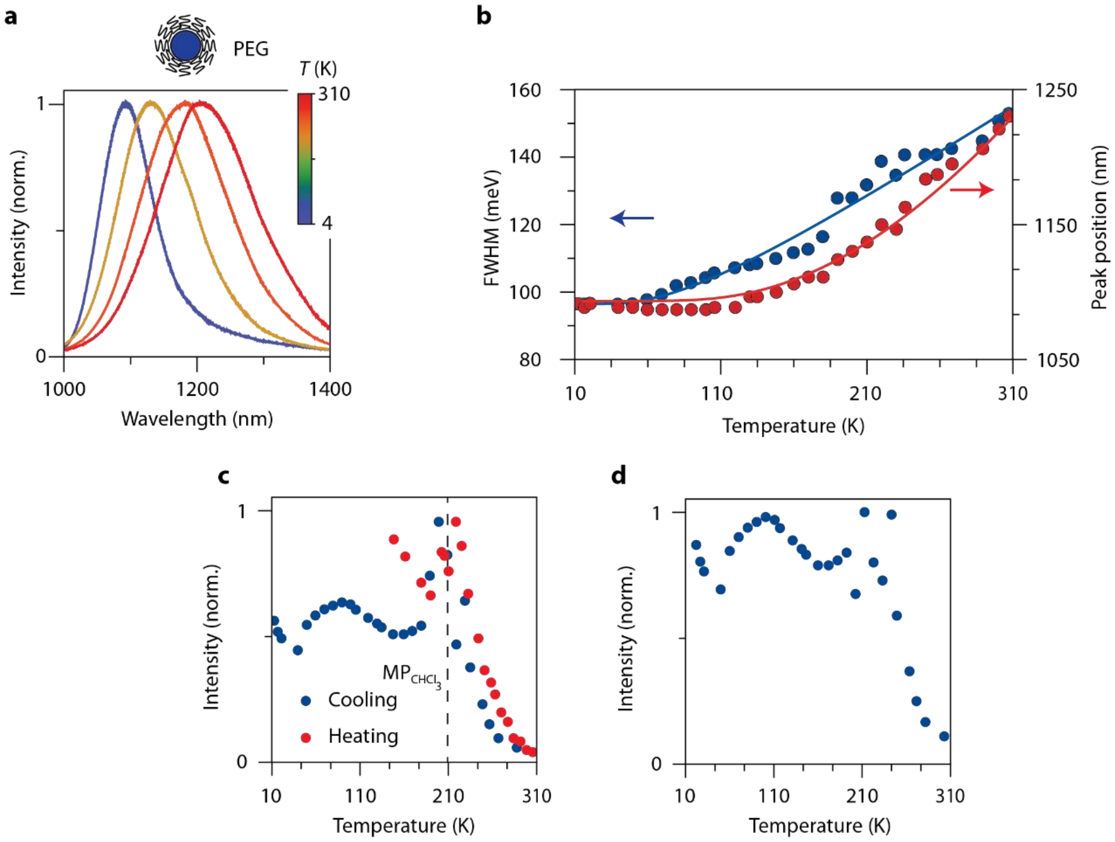

Figure S5 | Temperature-dependent emission spectroscopy on of Ag_2S-PEG NCs. (**a**) Emission spectra of Ag_2S-PEG NCs recorded at (blue to red) 13, 128, 220 and 289 K. (**b**) Plot of the FWHM and the peak position as a function of temperature for the Ag_2S-PEG NCs. The blue line is a fit with equation S.2 to the temperature vs. fwhm data. The red line is a fit of equation S.3 to the temperature vs. peak position data. Fit outcomes can be found in table S3 (**c**) Integrated photoluminescence intensity plotted as a function of temperature recorded while cooling down (blue) and while warming up (red). The dashed line marks the melting temperature of $CHCl_3$. (**d**) Same as in panel **c** but corrected for the intensity jump created by the solidification of $CHCl_3$.

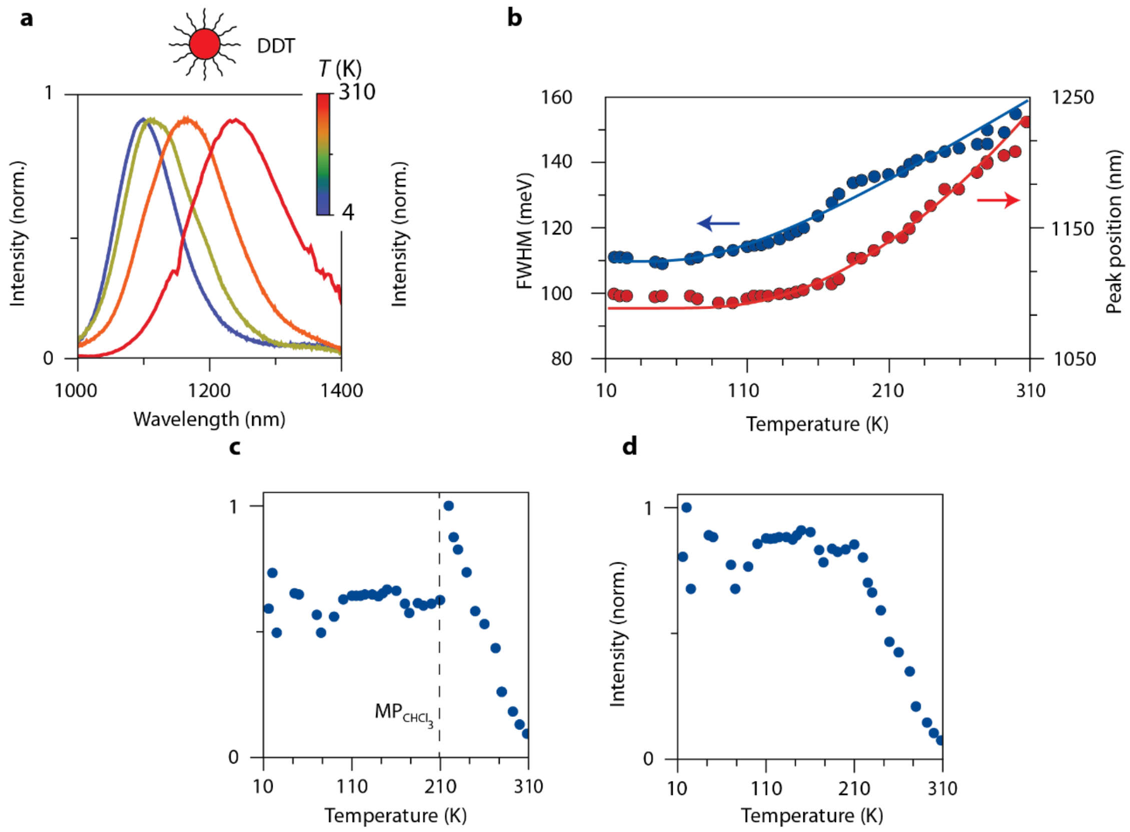

Figure S6 | Temperature-dependent emission spectroscopy of Ag_2S-DDT NCs. (**a**) Emission spectra of Ag_2S-DDT NCs recorded at (blue to red) 13, 128, 220 and 289 K. (**b**) Plot of the fwhm and the peak position as a function of temperature for the Ag_2S-DDT NCs. The blue line is a fit with equation S.2 to the temperature vs. fwhm data. The red line is a fit of equation S.3 to the temperature vs. peak position data. Fit outcomes can be found in table S3 (**c**) Integrated photoluminescence intensity plotted as a function of temperature recorded while cooling down (blue). The dashed line marks the melting temperature of $CHCl_3$. (**d**) Same as in panel **c** but corrected for the intensity jump created by the solidification of $CHCl_3$.

Changes in the main text: In the discussion of Figure 2 we refer to the further analysis of electron-phonon coupling in the Supporting information: 'Analysis of the temperature dependent broadening of the emission band and the peak shift confirms the strong electron-phonon coupling for emission transition (Supporting Information section S6).'

7. The measured Ag2S NCs underwent sonication procedure in CHCl3 which may resulting in chlorine-passivation. Whether the data and conclusions for Ag2S NCs obtained using the passivated NC are persuasive? In addition, the conclusion for "perfectly passivated NCs" may be inappropriate. Will other better Cl-passivated Ag2S NCs with higher PLQY (Adv. Optical Mater. 2022, 10, 2102806) lead to different conclusions?

Our response: The presence of Cl ions on the surface of sonicated nanoparticles has been studied by our group in a previous work (https://doi.org/10.1021/acsami.1c19344) and after a thorough study by means of XPS and EDS we were unable to observe the presence of Cl ions and therefore we rule out this hypothesis. XPS spectra are shown below for Ag_2S-DDT (black) and Ag_2S-PEG (red) nanoparticles, showing that in the Cl2p energy binding region no signal was detected, ruling out the presence of Cl within the structure of the synthesize NPs. The high quantum yield of almost 50% in the Adv. Optical Mater. publication mentioned may be

overestimated. Details on the quantum yield measurements are sparse in this report and the short emission life time (256 ns) is not consistent with such a high quantum yield and microseconds radiative lifetimes reported for the Ag_2S emission. Quantum yield measurements in the infrared are notoriously challenging. Other papers in the literature have not reproduced such high quantum yields and the highest yields reported are ~10%, consistent with our observation of intrinsic luminescence quenching of the trapped exciton emission in Ag_2S NCs.

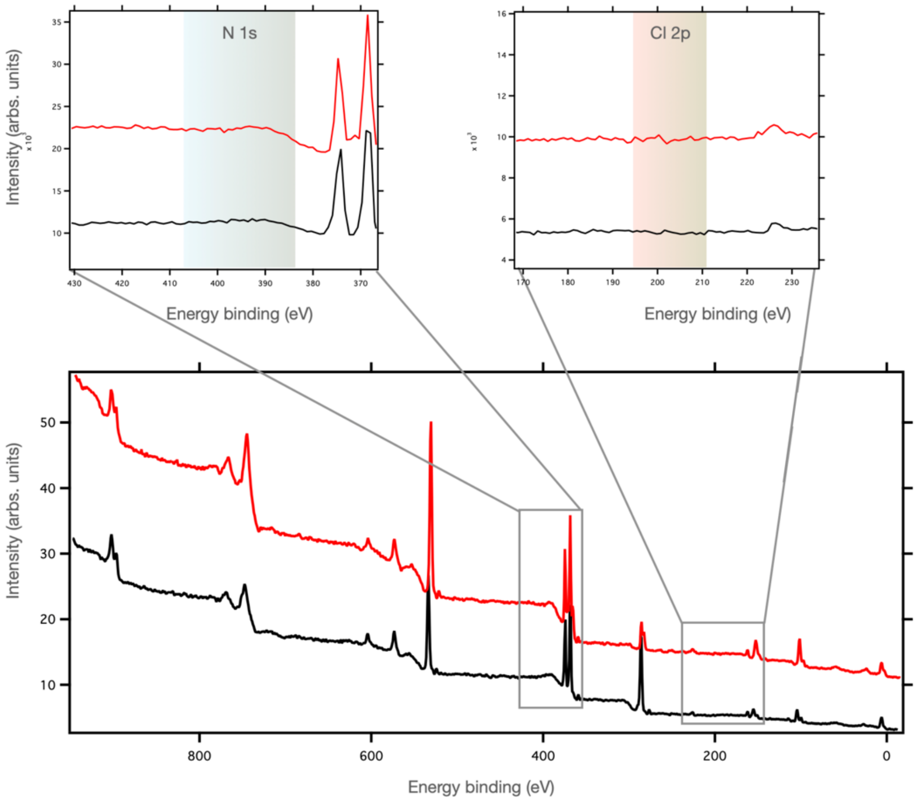

Additional Questions:
Urgency: **Moderate**
Significance: **High**
Novelty: **High**
Scholarly Presentation: **High**
Is the paper likely to interest a substantial number of physical chemists, not just specialists working in the authors' area of research?: **Yes**

jz-2024-01439q.R2

Name: Peer Review Information for "New Insights in Luminescence and Quenching Mechanisms of Ag₂S Nanocrystals through Temperature-Dependent Spectroscopy"

Second Round of Reviewer Comments

Reviewer: 1

Comments to the Author

The authors have addressed the issues.

Reviewer: 2

Comments to the Author

My concerns have been addressed.

Author's Response to Peer Review Comments:

Dear Editor,

Thank you for accepting our manuscript. The revisions requested by the editorial office have been implemented.
